# Naturally Segregating Variation at *Ugt86Dd* Contributes to Nicotine Resistance in *Drosophila melanogaster*

**DOI:** 10.1534/genetics.117.300058

**Published:** 2017-07-26

**Authors:** Chad A. Highfill, Jonathan H. Tran, Samantha K. T. Nguyen, Taylor R. Moldenhauer, Xiaofei Wang, Stuart J. Macdonald

**Affiliations:** * Department of Molecular Biosciences, University of Kansas, Lawrence, Kansas 66047; † Center for Computational Biology, University of Kansas, Lawrence, Kansas 66047

**Keywords:** xenobiotics, CRISPR, functional validation, QTL mapping, RNAi

## Abstract

Identifying the sequence polymorphisms underlying complex trait variation is a key goal of genetics research, since knowing the precise causative molecular events allows insight into the pathways governing trait variation. Genetic analysis of complex traits in model systems regularly starts by constructing QTL maps, but generally fails to identify causative sequence polymorphisms. Previously we mapped a series of QTL contributing to resistance to nicotine in a *Drosophila melanogaster* multiparental mapping resource and here use a battery of functional tests to resolve QTL to the molecular level. One large-effect QTL resided over a cluster of UDP-glucuronosyltransferases, and quantitative complementation tests using deficiencies eliminating subsets of these detoxification genes revealed allelic variation impacting resistance. RNAseq showed that *Ugt86Dd* had significantly higher expression in genotypes that are more resistant to nicotine, and anterior midgut-specific RNA interference (RNAi) of this gene reduced resistance. We discovered a segregating 22-bp frameshift deletion in *Ugt86Dd*, and accounting for the InDel during mapping largely eliminates the QTL, implying the event explains the bulk of the effect of the mapped locus. CRISPR/Cas9 editing of a relatively resistant genotype to generate lesions in *Ugt86Dd* that recapitulate the naturally occurring putative loss-of-function allele, leads to a large reduction in resistance. Despite this major effect of the deletion, the allele appears to be very rare in wild-caught populations and likely explains only a small fraction of the natural variation for the trait. Nonetheless, this putatively causative coding InDel can be a launchpad for future mechanistic exploration of xenobiotic detoxification.

A principal goal of research on the genetics of complex traits is to identify the precise sequence polymorphisms responsible for phenotypic variation. This is either carried out directly in natural populations or in laboratory mapping panels derived from a sample of naturally derived chromosomes. The pursuit of causative alleles is open to criticism (Rockman 2012), since with finite power it is necessarily the case that experimentally identified causative variants represent a biased set of the complete collection of sites impacting variation. Nonetheless, genomewide mapping studies have provided important contributions to our understanding of polygenic trait variation. Robust, replicable associations from unbiased genomewide association studies (GWAS) have provided considerable insight into the pathways underlying disease risk (Hirschhorn 2009; Visscher *et al.* 2012). In addition, the genes and molecular lesions contributing to an array of crop domestication traits have been identified (Doebley *et al.* 2006; Gross and Olsen 2010), providing detail on the specific molecular differences between modern crop plants and their wild progenitors and an understanding of the nature of the selection applied.

In model organisms, the hunt for causative variation commonly begins with linkage-based QTL mapping. Whether initiated with two parental strains (Lander and Botstein 1989) or, more recently, with several founders (Kover *et al.* 2009; Churchill *et al.* 2012; King *et al.* 2012b; Threadgill and Churchill 2012), such mapping designs have tremendous power to find QTL, and in some cases have led to the identification of specific polymorphisms contributing to trait variation (*e.g.*, Long *et al.* 2000; Deutschbauer and Davis 2005; Bendesky *et al.* 2011; Cook *et al.* 2016; Linder *et al.* 2016). These variants facilitate a deeper understanding of specific biomedically relevant traits and collectively add to a fundamental appreciation of complex trait variation and its maintenance in populations. However, with the possible exception of yeast, where one can test vast numbers of recombinants and minimize the statistical challenges associated with finding small-effect variants at high resolution (Ehrenreich *et al.* 2010), it is quite clear that linkage-based genomewide mapping for most complex traits in most systems does not result in the identification of a causative mutation.

The difficulty with moving from QTL to causative site is compounded if traits are highly polygenic. Cornforth and Long (2003) have shown by simulation that standard QTL mapping approaches are unable to discriminate between a single QTL of large effect and a series of very closely linked QTL that each have independent effects. That such collections of adjacent causative sites exist is amply demonstrated by fine mapping studies that succeed in “fractioning” a single QTL into multiple causative loci (Pasyukova *et al.* 2000; Steinmetz *et al.* 2002; Kroymann and Mitchell-Olds 2005), from elegant work showing that multiple alleles combine to yield the major effect of the *Adh* gene on alcohol dehydrogenase expression in flies (Stam and Laurie 1996) and from recent work in multiparental mapping panels showing that QTL frequently appear to be generated by more than one segregating site (Baud *et al.* 2013; King *et al.* 2014). Thus, to follow up QTL mapping and identify functional allelic variation at the sequence level, additional molecular and functional tests are typically required.

In Marriage *et al.* (2014) we described initial genetic dissection of nicotine resistance in *D. melanogaster* larvae, expanding on studies that have identified genes and transcripts influencing related nicotine resistance traits in flies (Passador-Gurgel *et al.* 2007; Li *et al.* 2012). Nicotine is an ecologically and biomedically interesting toxin for three reasons. It is produced by a number of plants, most notably *Nicotiana* species, as a defense against herbivory (Glendinning 2002; Steppuhn *et al.* 2004). Nicotine was also commonly used as an agricultural pesticide in the middle of the 20th century (Shepard 1951; Metcalf 1955), although it has since been supplanted by other insecticides, including the chemically related neonicotinoids. Finally, nicotine continues to be responsible for large numbers of human deaths due to its addictive properties (Jha *et al.* 2013).

Marriage *et al.* (2014) used lines from the *Drosophila* Synthetic Population Resource (DSPR), a set of inbred strains derived from a multiparental, advanced generation intercross (King *et al.* 2012b), and mapped a number of nicotine resistance QTL. Two loci, Q1 on chromosome 2L and Q4 and 3R, had large effects on phenotype, explaining 8.5 and 50.3% of the broad sense heritability, respectively. Both intervals contained genes encoding detoxification enzymes, with Q1 encompassing two cytochrome P450 genes, and Q4, a set of 10 UDP-glucuronosyltransferase (or UGT) genes. These genes are strong *a priori* candidates to contribute to xenobiotic resistance, offering the possibility of identifying the underlying causative alleles. Our goal here was to provide evidence that one or more of these genes directly contributes to variation in resistance, employing fine mapping, expression profiling, tissue-specific RNAi, overexpression analyses, and CRISPR/Cas9 genome editing. Our data point to a single 22-bp InDel event in a coding region of *Ugt86Dd* as a major factor contributing to the previously mapped Q4 locus.

## Materials and Methods

### Larval nicotine resistance assay

We used the same assay described in Marriage *et al.* (2014), except here we tested larvae in narrow polystyrene vials rather than in plates. Briefly, flies were allowed to lay eggs on a cornmeal–yeast–molasses medium containing 0.5% activated charcoal, supplemented with a small amount of active yeast paste to elicit egg laying. First instar larvae were collected and placed either on no-drug media or on media containing 0.18 μl/ml nicotine (N3876; Sigma). No-drug and nicotine media were always prepared the day prior to larval collection to minimize variation due to nicotine breakdown. Replicate assay vials contained 30 first instar larvae in nearly all cases (Supplemental Material, File S1), and the phenotype for each replicate vial is given as the fraction of larvae that ultimately emerge as adults. For every genotype tested, we set up several egg-laying vials, and the mean phenotype is given as the average of multiple replicate assay vials.

All animals were reared and tested at 25° and 50% relative humidity on a 12-hr-light/12-hr-dark cycle. Those test animals that were the result of crosses were generated by pairing 10 virgin females with four to five males over several replicate vials. Prior to initiating such crosses, parental flies were allowed to recover from CO_2_ anesthesia for 24 hr.

### Chromosome substitution

Marriage *et al.* (2014) identified four autosomal QTL contributing to nicotine resistance and found that for each QTL, recombinant inbred lines (RILs) harboring the A4 founder allele were on average more resistant to nicotine than those harboring the A3 allele (see figure 3 in Marriage *et al.* 2014). To broadly examine the phenotypic effects of the A3 and A4 alleles, we generated the chromosome substitution lines A4/A3; A4; A3 and A3/A4; A3; A4 (chromosomes are listed in the order X/Y; 2; 3). We directly compared these substitution genotypes to inbred strains A3 and A4 and carried out intercrosses among strains to explore dominance. For each genotype, we measured the phenotype using two replicate no-drug vials to test for variation in overall viability and six replicate nicotine-containing vials.

To generate chromosome substitutions, the following strains were obtained from the Bloomington *Drosophila* Stock Center (BDSC): the double-balancer strain 8316 (w^1118^; wg^Sp-1^/CyO; sens^Ly-1^/TM6B, Tb^1^), the GFP-marked chromosome 2 balancer strain 5702 [w^1^; sna^Sco^/Cyo, Gal4-Hsp70, upstream activating sequence (UAS)-GFP], and the GFP-marked chromosome 3 balancer strain 5704 (w^1^; Sb^1^/TM3, Gal4-Hsp70, UAS-GFP, y^+^ Ser^1^).

### Quantitative complementation tests

To test for the effects of allelic variation between strains A3 (relatively susceptible to nicotine) and A4 (relatively resistant to nicotine) at particular genomic regions and at certain genes, we carried out quantitative complementation tests. Virgin females from A3 and A4 were each crossed to males from a series of mutation-containing lines, along with their co-isogenic wild-type control lines, allowing the production of four types of F_1_ progeny per target mutation (A3/mutation, A3/control, A4/mutation, and A4/control). Phenotypes were based on a total of 2–8 replicate no-drug vials, and 8–18 replicate nicotine-containing vials for each genotype. We employed the following model for analysis: *y* = *μ* + *S* + *D* + (*S* × *D*), where *S* is the effect of founder strain (A3 or A4), *D* is the effect of the mutation (comparing either a deficiency or an insertion to wild type), and *S* × *D* is the interaction. A significant *S* × *D* term indicates a failure to complement (A3 and A4 functionally differ at the locus under test).

Exelixis (Parks *et al.* 2004) and Bloomington Stock Center (BSC) (Cook *et al.* 2012) deficiencies were obtained from the BDSC, specifically 7497 [w^1118^; Df(2L)Exel6011/CyO], 7957 [w^1118^; Df(3R)Exel7306/TM6B, Tb^1^], 7958 [w^1118^; Df(3R)Exel8152/TM6B, Tb^1^], and 26545 [w^1118^; Df(2L)BSC693/SM6a], along with the co-isogenic control strain 6326 (w^1118^). We additionally obtained the DrosDel (Ryder *et al.* 2004) deficiency 9083 [w^1118^; Df(3R)ED5506/TM6C, cu^1^ Sb^1^] and its co-isogenic control 5905 (also w^1118^ but not necessarily otherwise identical to 6326). All deficiencies are maintained over balancers, and since we must discriminate deficiency-containing and balancer chromosomes in larvae, prior to use we substituted all native balancers with GFP-marked versions derived from BDSC stocks 5702 and 5704 (see above). Finally, we obtained several Minos-based insertional mutants (Metaxakis *et al.* 2005), all of which were generated in the 5905 background: 23530 [w^1118^; Mi{ET1}4Cyp28d1^MB03293^], 23587 [w^1118^; Mi{ET1}Cyp28d2^MB02776^], 24834 [w^1118^; Mi{ET1}Ugt86Dj^MB04890^], and 27861 [w^1118^; Mi{ET1}Ugt86Dh^MB11311^].

### RNAseq to identify nicotine resistance genes

We selected six (12) lines with relatively high (low) resistance (File S2) from the set of DSPR pB2 RILs that were previously scored for nicotine resistance (Marriage *et al.* 2014). Strains were raised as described above: 30 first instar larvae from each were placed on no-drug or nicotine-supplemented media for 2 hr, after which larvae were flash frozen in liquid nitrogen and stored at −80°. We isolated RNA from each sample using TRIzol reagent (15596018; Thermo Fisher Scientific, Waltham, MA) and purified using RNeasy columns (74104; Qiagen, Valencia, CA). We then pooled equal amounts of total RNA from each sample to generate four RNA pools (high/nicotine, low/nicotine, high/no-drug, low/no-drug), generated an Illumina TruSeq unstranded messenger RNA (mRNA) library from each sample, and combined libraries to sequence over a single HiSeq2500 lane and generate single-end 100-bp reads [University of Kansas (KU) Genome Sequencing Core]. Reads were quality trimmed using scythe (v0.991, github.com/vsbuffalo/scythe) and sickle (v1.200, github.com/najoshi/sickle), assembled to the *D. melanogaster* reference genome [National Center for Biotechnology Information (NCBI) Build 5.3, ccb.jhu.edu/software/tophat/igenomes.shtml] using TopHat v2.0.10 (Trapnell *et al.* 2009; Kim *et al.* 2013), and significant expression differences between relevant pooled samples were identified using Cufflinks v.2.2.1 (Trapnell *et al.* 2010, 2013).

### RNAi knockdown

We employed the binary Gal4-UAS RNAi system to knock down expression of several candidate detoxification genes ubiquitously and specifically in a number of tissues. We crossed males from each Gal4 driver strain to females containing a UAS-RNAi transgene, or to appropriate control females, and assayed F_1_ Gal4-UAS-RNAi progeny. For each genotype we phenotyped 3–5 replicate no-drug vials and 8–10 replicate nicotine vials.

We obtained UAS-RNAi transgene-carrying stocks from the Vienna *Drosophila* Resource Center (VDRC) (Dietzl *et al.* 2007). The “GD” library strains harbor *P*-element-derived UAS transgenes, while the “KK” library strains harbor phiC31-based transgenes, where all transgenes reside at the same landing site. Specifically, we use VDRC UAS-RNAi strains 6016 (GD, UAS-RNAi-*Ugt86Dd*), 7870 (GD, UAS-RNAi-*Cyp28d1*), 7868 (GD, UAS-RNAi-*Cyp28d2*), 100353 (KK, UAS-RNAi-*Ugt86Dd*), 102626 (KK, UAS-RNAi-*Cyp28d2*), and 110259 (KK, UAS-RNAi-*Cyp28d1*). We directly compare genotypes containing these transgenes to the appropriate control strains 60000 (GD control strain) and 60100 (KK control strain). In addition, we obtained the Transgenic RNAi Project (TRiP) (Perkins *et al.* 2015) strain 53892 (UAS-RNAi-*Cyp28d1*) and its co-isogenic control (36304) from the BDSC.

We drove RNAi in all cells and all timepoints using *Act5C*-Gal4 [derived from BDSC 3954 (y^1^ w*; P{Act5C-GAL4}17bFO1/TM6B, Tb^1^)], after first substituting the third chromosome balancer in this strain for the GFP-marked version derived from BDSC 5704 (see above). To drive RNAi in specific groups of malpighian tubule cells, we employed a series of strains obtained from Julian Dow and Shireen Davies (University of Glasgow): c42-Gal4, c710-Gal4, c724-Gal4, and uro-Gal4 (Rosay *et al.* 1997; Sozen *et al.* 1997; Terhzaz *et al.* 2010). We obtained the anterior midgut Gal4 driver 1099 from Nicolas Buchon (flygut.epfl.ch). Finally, we obtained a number of strains expressing Gal4 in various parts of the gut from the BDSC, specifically strains 1967 [y^1^ w*; P{GawB}34B], 30828 [w*; P{GawB}Alp4^c232^], 30844 [w*; P{GawB}c601^c601^], and 43656 [w*; P{Scr-GAL4.4}1-3].

### Overexpression of *Ugt86Dd*

A gain-of-function analysis was performed for *Ugt86Dd* by creating UAS-*Ugt86Dd* overexpression strains. We PCR amplified the gene from both A3 and A4, and simultaneously added *attB* sites, using the primers 5′-GGGGACAAGTTTGTACAAAAAAGCAGGCTTACAACATGAGATTATTAACTGTGATCGCGA-3′ and 5′-GGGGACCACTTTGTACAAGAAAGCTGGGTCCTAATGTTTCTTAAGCTTATCAG-3′. Following the manufacturer’s protocol (PCR Cloning System with Gateway Technology, 12535029; Thermo Fisher Scientific) we cloned the PCR products into the pDONR221 vector, creating entry clones via BP recombination reactions. Clones were Sanger sequenced using M13 primers to verify the sequence and direction of the insert. The destination vector pUASg.attB was donated by Johannes Bischof (Bischof *et al.* 2013) and used in combination with the LR reaction (Gateway LR Clonase, 11791020; Thermo Fisher Scientific) to generate both pUASg.Ugt86Dd-A3.attB and pUASg.Ugt86Dd-A4.attB expression clones. Expression clones were Sanger sequenced using primer hsp-GW-F (5′-GCAACTACTGAAATCTGCCAAG-3′) to verify the direction of the insert. To generate fly stocks we injected BDSC stock 24749 [y^1^ M{vas-int.Dm}ZH-2A w*; M{3xP3-RFP.attP}ZH-86Fb] with the expression plasmids at 0.510-0.515μg/μl (BestGene, Chino Hills, CA). We obtained one A3 transformant line (UAS-*Ugt86Dd*^A3^), and five A4 transformants [UAS-*Ugt86Dd*^A4(1)^ to UAS-*Ugt86Dd*^A4(5)^], that are each homozygous for the transgene-containing chromosome 3. These strains were utilized in conjunction with tissue-specific Gal4 drivers (above) to explore the effects of *Ugt86Dd* overexpression. For each genotype, we tested two to three replicate no-drug vials and six replicate nicotine vials.

### PCR genotyping assay for *Ugt86Dd* InDel variant

We identified a 22-bp InDel variant in a protein-coding region of exon two of *Ugt86Dd* and developed a PCR assay to genotype it. Primers 5′-CGCTTTTGCTCAGCATTTTA-3′ and 5′-ATATGTGGCAGGTGAACGAA-3′ amplify 219-bp and 197-bp products in the presence of the insertion and deletion allele, respectively. PCR cycling conditions were 95° for 2 min, 35 cycles of 95° for 20 sec, 57° for 25 sec, and 72° for 30 sec, with a final 2-min extension at 72°. Products were sized on 2% gels run at low voltage for up to 2 hr.

### QTL mapping

Marriage *et al.* (2014) detected a nicotine resistance QTL—Q4—that encompasses *Ugt86Dd* using the DSPR. To test whether the 22-bp InDel in *Ugt86Dd* might contribute to the QTL effect, we first established the allelic status of the DSPR founder lines via PCR, finding that founders A3, AB8, B6, and B7 possess the deletion (Figure S1). Next, we employed the estimated founder genotype probabilities for the DSPR RILs (King *et al.* 2012b) to determine the InDel status of each RIL (File S3).

Methods for QTL mapping in the DSPR are described in King *et al.* (2012a,b). We employed the R (r-project.org) packages *DSPRqtl*, *DSPRqtlDataA*, and *DSPRqtlDataB* for mapping (FlyRILs.org), analyzed the pA and pB panels of RILs separately, and—in addition to the InDel covariate—included a covariate accounting for the subpopulation from which the RIL was derived (pA1, pA2, pB1, and pB2). Note that in the analyses conducted here, we only employed RILs for which we had high confidence in the founder haplotype at the *Ugt86Dd* gene, so sample sizes are marginally reduced compared to those employed in Marriage *et al.* (2014), and estimates of the QTL effect are slightly different than previously reported. To establish significance of mapped QTL, we generated genomewide 5% significance thresholds via 1000 permutations (Churchill and Doerge 1994).

### Testing the effect of the InDel variant in the *Drosophila* Genetic Reference Panel

Our PCR assay revealed that seven strains from the *Drosophila* Genetic Reference Panel (DGRP) (Mackay *et al.* 2012; Huang *et al.* 2014) were homozygous for the deletion (BDSC nos. 25176, 25177, 25206, 28185, 28199, 28213, and 37525). We also confirmed that the following seven strains were homozygous for the much more common insertion allele (25174, 25200, 28160, 28197, 28226, 28239, and 28295). We created a pair of populations, one fixed for the deletion and one fixed for the insertion, by carrying out all possible crosses between the seven founding strains (10 virgin females × 10 males), collecting 10 F_1_ progeny of each sex from each cross, and combining into a 1-gallon population bottle. Both populations were maintained for seven generations, at which point we phenotyped each population using 20 replicate nicotine-containing vials.

### *Ugt86Dd* CRISPR/Cas9 editing

We used the CRISPR Optimal Target Finder (tools.flycrispr.molbio.wisc.edu/targetFinder) to identify an appropriate guide RNA (gRNA) target sequence within *Ugt86Dd* (Gratz *et al.* 2014), specifically targeting the site of the 22-bp InDel. The target sequence 5′-TCACTACGAAGTCATTGTGGAGG-3′ matches the sequence of A4 and was used to generate a gRNA that leads to double-strand breaks within the insertion sequence (Figure S1). We purchased the 5′-phosphorylated sense and antisense oligos 5′- CTTCGTCACTACGAAGTCATTGTGG-3′ and 5′-AAACCCACAATGACTTCGTAGTGAC-3′, annealed, and followed the protocol from Gratz *et al.* (2013) to clone the annealed oligos into the pU6–BbsI–chiRNA plasmid (Addgene, Cambridge, MA; plasmid 45946). Transformants were verified via Sanger sequencing.

Our goal was to compare the phenotypes of inbred strains differing for wild-type and mutant *Ugt86Dd* alleles in an otherwise genetically identical background. Preliminary sequencing revealed that chromosome 3 for the *vasa*-Cas9 BDSC strain 55821 [y^1^ M{vas-Cas9.RFP-}ZH-2A w^1118^] is not homozygous. Thus, we used a series of standard fly crosses with balancers to substitute chromosome 3 from founder strain A4 into 55821, creating a strain homozygous for *vasa*-Cas9 on the X and A4 on chromosome 3 (the state of chromosome 2 is unknown, but is likely heterozygous).

The gRNA plasmid was injected into 300 embryos from our custom *vasa*-Cas9; ; A4 injection strain at 250 ng/μl (BestGene) yielding 89 G_0_ progeny, 57 of which yielded progeny after being individually crossed to one to two animals from BDSC 5704 (see above). Where possible we collected 10 F_1_ balanced progeny from each G_0_ cross, and individually crossed to one to two animals from BDSC 5704. For crosses containing a mutation (see below), and for some that did not, we collected balanced F_2_ progeny of both sexes to establish stocks, removing the balancer in subsequent generations to create strains carrying homozygous third chromosomes. These strains can and do segregate on the X and chromosome 2.

After each F_1_ cross produced eggs, we genotyped the F_1_ animal for the presence of a CRISPR/Cas9-induced mutation using T7 endonuclease [New England Biolabs (NEB), Ipswich, MA; M0302L]. Briefly, following single-fly DNA isolation (Puregene Cell and Tissue Kit, 158388; Qiagen) we PCR amplified a region surrounding the gRNA target site using oligos 5′-ACGCTTTTGCTCAGCATTTT-3′ and 5′-GGCTGGGGATACCATTTCTT-3′ (95° for 2 min, 35 cycles of 95° for 20 sec, 57° for 25 sec, and 72° for 30 sec, with a final 2-min extension at 72°). Subsequently 10 μl of PCR product was mixed with 7 μl of molecular grade water and 2 μl of NEB buffer 2 (B7002S), incubated at 95° for 5 min in a heat block, and allowed to slowly cool to room temperature for ∼2 hr. We then added 1 μl of T7 endonuclease to each reaction, incubated at 37° for 15 min, added 2 μl of 0.25 mM EDTA to stop the reaction, and immediately loaded the entire reaction volume into a 1.5% agarose gel. We also ran DNA from A4 and from the F_1_ progeny of A3 × A4 crosses (which are heterozygous for the 22-bp InDel) through this genotyping pipeline as negative and positive controls, respectively.

Editing led to 16 strains with homozygous third chromosomes carrying independent mutations in *Ugt86Dd* (File S4). Simultaneously, we generated seven strains containing unedited homozygous third chromosomes. Each of the 23 strains created was phenotyped using 5–6 replicate no-drug and nicotine-containing vials. For two edited and one unedited genotype, we substituted the edited third chromosome into the A4 background, allowing a direct comparison of mutated and wild-type versions of *Ugt86Dd*. Each of these strains—A4-*Ugt86Dd^Del1^*, A4-*Ugt86Dd^Del11^*, and A4-*Ugt86Dd^wt^* (File S4)—was tested directly as an inbred genotype, and as a heterozygote following crosses to A4, using 10 replicate no-drug and nicotine-containing vials.

### Estimating the frequency of the InDel in nature

We can identify the two alleles at the short InDel directly from reads resulting from next-generation sequencing of pooled population samples of *D. melanogaster*, thereby estimating allele frequency. We retrieved raw FASTQ files from 13 wild-caught, resequenced population samples (Bergland *et al.* 2014) via the NCBI Sequence Read Archive (SRA) (BioProject accession PRJNA256231), and used a Unix grep procedure to count the number of raw reads containing the insertion and deletion sequences. We ensured that instances of a sequence occurring in both reads of a single paired-end fragment were counted only once. The insertion-specific sequence was taken as the central 20-bp (*i.e.*, ATTGTGGAGGACATTCATCG) of the 22-bp InDel and its reverse complement. The deletion-specific sequence was taken as the 10-bp either side of the 22-bp InDel (*i.e.*, GAACGAATTCACTTCGTAGT), and its reverse complement. These query sequences match all 15 inbred DSPR founder strains (Figure S1).

### Data availability

All raw phenotypes are presented in File S1. Raw RNAseq reads are available from the NCBI SRA (accession no. SRP102254).

## Results

### Large, dominant effect of chromosome 3 on nicotine resistance

**Figure 1:**
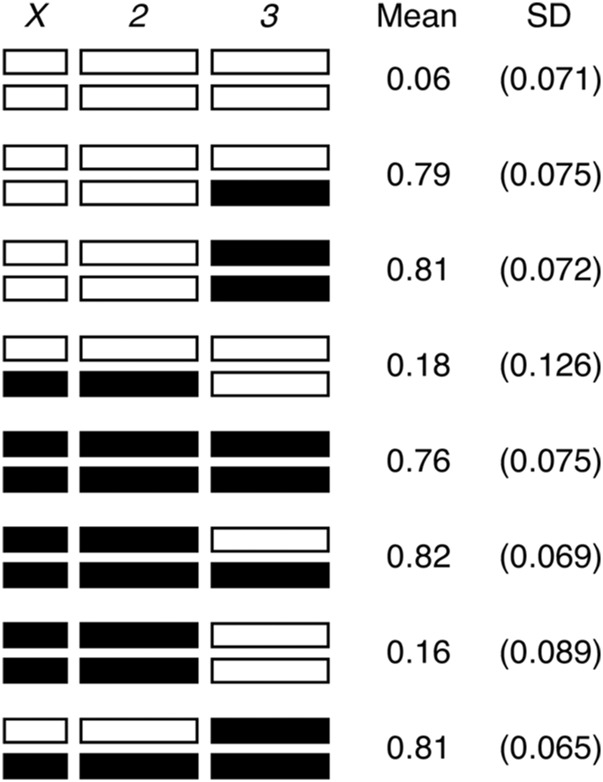
Effects of chromosome substitution on nicotine resistance. Chromosomes derived from DSPR founder A3 are shown in white, and those from A4 are shown in black. Nicotine resistance for each genotype was tested across six replicate vials, and we performed all possible pairwise *t*-tests among genotypes. The three low resistance genotypes are statistically indistinguishable, as are the five high resistance genotypes, while all pairs of low and high resistance genotypes are significantly different (*P* < 0.0001 in all cases). The viability of each genotype was tested twice under no-drug conditions, and all genotypes showed a mean viability of >0.9.

Marriage *et al.* (2014) identified four autosomal QTL contributing to nicotine resistance in the pA DSPR population. Three QTL on chromosome 2 (Q1–Q3) individually contributed 4.6–8.5% to the broad sense heritability of the trait, and one QTL on chromosome 3 (Q4) contributed 50.3% to the heritability. By estimating the effects associated with each founder allele at each mapped QTL, it appeared that in all cases, carrying an A4 allele at the QTL led to greater resistance than carrying an A3 allele. To confirm this, we tested various chromosome substitution genotypes and observed that moving a single third chromosome from A4 into an otherwise A3 background led to a marked increase in resistance compared to A3 (*P* < 10^−8^, Figure 1). This implies that A3 and A4 do harbor different alleles at functional variants within Q4, and that the effect of the A4 chromosome is dominant.

In contrast to the large effect of chromosome 3, the combined effects of the X and chromosome 2 on nicotine resistance are relatively small; A genotype with the A3 third chromosome homozygous in an otherwise A4 background is only marginally more resistant than founder A3 (*P* = 0.07, Figure 1). This result confirms the smaller phenotype effects estimated at the second chromosome QTL by Marriage *et al.* (2014).

### Fine mapping nicotine resistance QTL

One strategy to fine map QTL is to employ quantitative complementation tests, either using deletions that remove tens to hundreds of genes (Pasyukova *et al.* 2000), or loss-of-function mutant alleles of plausible candidate genes (Long *et al.* 1996). We crossed the A3 and A4 founders, which differ in nicotine resistance, to a series of mutation-carrying strains and their corresponding co-isogenic, mutation-free control strains, generating four types of progeny (A3/mutation, A3/control, A4/mutation, and A4/control). We then used a statistical test that estimates the effects of founder and mutation, and the interaction between these factors. A significant interaction—a quantitative failure to complement—implies the effects of founder alleles are different in the mutant and control backgrounds, and thus suggests the founders segregate for functional variation at the tested deficiency/gene.

We employed two small deficiencies that deleted overlapping regions of the Q1 region, one of which eliminates both of the P450 genes within the interval, *Cyp28d1* and *Cyp28d2*, and one of which deletes only *Cyp28d1*. Since the deletions remove a total of 11–13 genes, including three additional P450s not within the Q1 interval, we also employed a pair of Minos insertions (Metaxakis *et al.* 2005) that insert within coding exons of *Cyp28d1* and *Cyp28d2*, likely disrupting gene function. In all four cases, there was a highly significant founder × mutant interaction (*P* < 10^−5^, Figure S2) with the difference between A3 and A4 being much greater in the mutant background. Collectively these results imply that both *Cyp28d1* and *Cyp28d2* show functional differences between A3 and A4 that confer effects on phenotype.

We carried out a similar experiment for the Q4 region, testing three overlapping deficiencies that individually delete 11–19 genes, including variable numbers of the UGT genes resident within Q4. All three deletions exhibit a significant quantitative failure to complement (*P* < 10^−6^, Figure S2). Since *Ugt86Dc* is the only gene deleted by all three deletions, a parsimonious explanation is that A3 and A4 have distinct nicotine resistance alleles at this gene. However, Marriage *et al.* (2014) showed that expression of this gene is reduced in A4 compared to A3, which is not the pattern one might expect under the assumption that *Ugt86Dc* leads to the enhanced nicotine resistance of A4. Additionally, that study provided evidence that the founder allelic effects at Q4 do not fall into two groups, as would be expected if a single causative gene was responsible for the QTL. Thus, the different deletions may be uncovering variants in independent genes that affect phenotype.

Finally, we tested a Minos element in a coding exon of *Ugt86Dj*, and one that resides within the 3′-UTR of one of the two isoforms of *Ugt86Dh*. (Mutations in a known background were available only for 2/10 of the UGT genes under Q4 when this experiment was conducted.) While we observed a significant founder × mutant interaction for both genes (*Ugt86Dj*, *P* < 0.001; *Ugt86Dh*, *P* < 0.01; Figure S2), in both cases the difference between the A3 and A4 alleles was greatest in the control background. These results suggest the effects are more likely due to epistasis than allelic failure to complement (see Mackay 2001; Geiger-Thornsberry and Mackay 2004), and do not provide strong evidence for the role of functional variation at *Ugt86Dh* or *Ugt86Dj* in resistance to nicotine.

### Candidate nicotine resistance genes via RNAseq

Under the assumption that some fraction of complex trait variation is regulatory in origin (Gibson and Weir 2005; Gilad *et al.* 2008; Gusev *et al.* 2014; Torres *et al.* 2014; Albert and Kruglyak 2015), we might expect to see changes in the expression of genes harboring causative loci. Marriage *et al.* (2014) used RNAseq of whole, first instar larvae from A3 and A4 tested under no-drug and nicotine-exposure conditions to attempt to resolve candidate genes. Here, we carried out a similar study employing mixed pools of RNA from relatively susceptible and relatively resistant pB DSPR RILs (File S2). Many genes showed differential expression between susceptible and resistant genotypes and/or between treatments (File S5), but those changes at loci within QTL intervals are of principal interest.

Considering those 34 genes within Q1, only *Cyp28d1* was differentially expressed at a nominal 5% level, showing an induction in expression on nicotine exposure in both susceptible (*P* < 10^−4^) and resistant animals (*P* = 0.001), confirming the results of Marriage *et al.* (2014). However, unlike our previous study, we found no change in expression between susceptible and resistant pB line pools that would indicate an allelic difference between genotypes at this gene. Since Q1 was not identified in the pB DSPR panel, it is possible there is no segregating variation that leads to expression variation within this panel, although we cannot discount the possibility we failed to capture any such variation in the small number of pB lines used for the current expression study (File S2).

At Q4, 5/10 UGT genes show a change in expression in at least one contrast, with two genes showing a change between susceptible and resistant genotypes; *Ugt35b* shows a slight increase in expression in the resistant genotypes under no-drug conditions (*P* < 0.05), while *Ugt86Dd* increases in expression in resistant animals under both no-drug and nicotine conditions (*P* < 10^−4^ and *P* = 0.001, respectively). Marriage *et al.* (2014) found no effect at *Ugt35b*, but did observe expression differences between A3 and A4 in the expression of *Ugt86Dd*. Since Q4 was identified in both the pA and pB populations, the similar change in expression between susceptible and resistant genotypes derived from the two panels implies *Ugt86Dd* is a strong candidate to harbor functional genetic variation contributing to resistance.

Marriage *et al.* (2014) identified a small peak in the LOD score profile in pB that encompasses the *Cyp6g1* gene, a gene shown to be associated with DDT (Daborn *et al.* 2002; Chung *et al.* 2007; Schmidt *et al.* 2010) and nicotine resistance (Li *et al.* 2012). However, we did not focus on the QTL in our previous study, given the modest LOD score at the peak and the relatively small number of pB RILs assayed. Here, we found a dramatic increase in expression of *Cyp6g1* between susceptible and resistant pB genotypes in both no-drug and nicotine conditions (*P* < 10^−4^), providing some additional support for the effect of this gene on nicotine resistance in the DSPR.

### Significant reduction in resistance following ubiquitous gene knockdown

**Figure 2:**
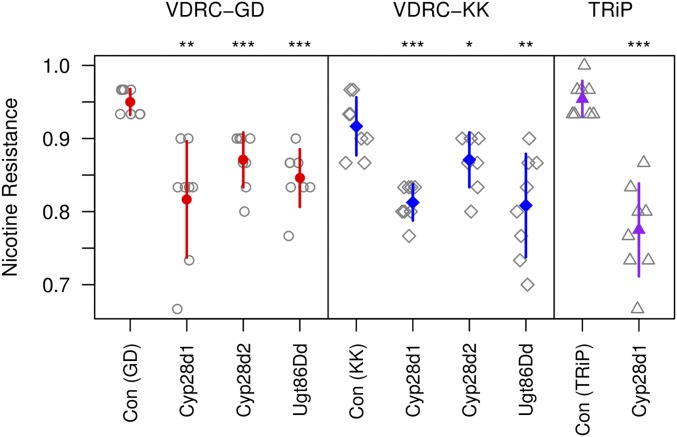
Effect of ubiquitous gene knockdown on nicotine resistance. We employed the Gal4-UAS-RNAi system to knock down the expression of three genes in all cells and at all timepoints via *Act5C*-Gal4. Each genotype was tested across eight replicate vials (raw data shown in gray symbols), and the mean (±1 SD) phenotype of each is shown with colored symbols/lines. Within each RNAi system (VDRC-GD, VDRC-KK, and TRiP) we tested the effect of each gene knockdown against its co-isogenic control using *t*-tests, and significance is highlighted by asterisks (* *P* < 0.05, ** *P* < 0.01, *** *P* < 0.001). All ubiquitous RNAi knockdowns reduce nicotine resistance. Note that the viability of each genotype was tested four to five times in no-drug conditions, and all genotypes showed a mean viability of >0.93.

Under Q1, the genes most likely to harbor functional variation impacting nicotine resistance are *Cyp28d1* and *Cyp28d2*. Under Q4, *Ugt86Dd* appears to be the strongest candidate based on RNAseq data. We knocked down the expression of these three genes using two to three different UAS transgenes per gene. In comparison with co-isogenic control strains, we see a robust reduction in resistance following knockdown of each gene (Figure 2). Notably, gene knockdown had no effect on viability under no-drug conditions; all strains showed phenotypes above 0.93 on no-drug food (compare to Figure 2), and there was no effect of gene knockdown under no-drug conditions (*P* > 0.4 for all tests). Thus, the effects of reducing expression of *Cyp28d1*, *Cyp28d2*, and *Ugt86Dd* appear to be specific to nicotine resistance.

### Knockdown of *Cyp28d1* and *Ugt86Dd* in the midgut reduces resistance

Data from FlyAtlas (Chintapalli *et al.* 2007; Robinson *et al.* 2013) indicates that *Ugt86Dd* expression is strongly enriched in the adult midgut, and in the adult and third instar larval malpighian tubules. In the same data set, *Cyp28d1* shows considerable among-tissue variation in expression, but has highest expression in the adult midgut and larval fat body, while *Cyp28d2* is strongly enriched in the larval midgut. All three genes additionally show variation in expression along the length of the midgut in third instar larvae (Harrop *et al.* 2014) and variation among adult midgut cell types (Dutta *et al.* 2015). These data show where the genes are expressed at high levels, but do not directly establish where they might act to metabolize nicotine. To help achieve this, we employed RNAi with a series of tissue-specific Gal4 drivers. Using two drivers that express Gal4 in the anterior region of the midgut, we saw that RNAi of *Cyp28d1* (in 5/6 cases) and *Ugt86Dd* (in 4/4 cases), but not *Cyp28d2* (in 0/4 cases), led to a significant reduction in resistance (Table 1). In contrast, a driver expressing Gal4 in the posterior region of the midgut showed no effect of any target gene (Table 1). These data imply that the anterior midgut is an important site of nicotine metabolism and is impacted by the action of the products of *Cyp28d1* and *Ugt86Dd*, genes showing expression variation between resistant and susceptible genotypes (above and Marriage *et al.* 2014). The lack of any apparent effect of *Cyp28d2* in the midgut (Table 1), in contrast to the effect observed following ubiquitous knockdown of the gene (Figure 2) might imply that *Cyp28d2* is required in a different, and untested tissue. Alternatively, the ubiquitous knockdown effect described above may be a false positive, which is known to occur at low rates in RNAi screens (*e.g.*, Mummery-Widmer *et al.* 2009; Schnorrer *et al.* 2010).

**Table 1:** Effect of midgut-specific gene knockdown on nicotine resistance

RNAi system	UAS genotype	Mean nicotine resistance (±1 SD)		
		Anterior midgut Gal4 (1099)	Anterior midgut Gal4 (43656)	Posterior midgut Gal4 (1967)
VDRC-GD	Control (GD)	0.95 ± 0.032	0.96 ± 0.032	0.83 ± 0.096
	*Cyp28d1*	0.90 ± 0.064*	0.92 ± 0.039*	0.88 ± 0.047^ns^
	*Cyp28d2*	0.92 ± 0.050^ns^	0.96 ± 0.033^ns^	0.89 ± 0.072^ns^
	*Ugt86Dd*	0.90 ± 0.070*	0.90 ± 0.025***	0.89 ± 0.093^ns^
VDRC-KK	Control (KK)	0.84 ± 0.063	0.95 ± 0.032	0.73 ± 0.099
	*Cyp28d1*	0.77 ± 0.086*	0.88 ± 0.050**	0.75 ± 0.085^ns^
	*Cyp28d2*	0.84 ± 0.052^ns^	0.93 ± 0.078^ns^	0.76 ± 0.124^ns^
	*Ugt86Dd*	0.77 ± 0.058*	0.89 ± 0.053**	0.75 ± 0.083^ns^
TRiP	Control (TRiP)	0.93 ± 0.060	0.94 ± 0.050	0.70 ± 0.069
	*Cyp28d1*	0.83 ± 0.073**	0.91 ± 0.054^ns^	0.78 ± 0.079^ns^

The Gal4-UAS-RNAi system was used to knock down expression of three genes in the midgut. The phenotype of each genotype was tested over 7–10 (mean = 9.5) replicate vials. Each gene knockdown was tested against the relevant co-isogenic control using a *t*-test (^ns^ *P* ≥ 0.05, * *P* < 0.05, ** *P* < 0.01, *** *P* < 0.001). The viability of each genotype was tested two to five times in no-drug conditions. These genotype means averaged 0.92, 24/30 of the genotypes have greater no-drug viability than nicotine viability, and for the other six genotypes no-drug viability was at most 4% less than that on nicotine food. These observations suggest the effects of these tissue-specific gene knockdowns are not a result of general defects in viability.

### Malpighian tubule knockdown of target detoxification genes enhances resistance

The malpighian tubules are an important site of xenobiotic metabolism in insects (Dow and Davies 2006; Yang *et al.* 2007), and we tested multiple Gal4 drivers that broadly express in the malpighian tubules, and also those that target specific cell types within the organ. RNAi against three genes (*Cyp28d1*, *Cyp28d2*, and *Ugt86Dd*) using drivers expressing Gal4 broadly in the malpighian tubules, and in one case additionally in the hindgut and ureter, revealed no major changes in resistance (Table 2). However, in nearly all cases, knockdown of these genes in either principal or stellate cells of the malpighian tubule led to a significant *increase* in resistance in comparison with control genotypes (Table 2). We note that under no-drug conditions, all genotypes associated with these malpighian tubule cell-specific tests show viabilities >0.93, implying the relative increase in resistance on gene knockdown is in response to the nicotine treatment.

**Table 2:** Effect of malpighian tubule RNAi on nicotine resistance

Gal4 driver	Mean nicotine resistance (±1 SD)			
	Control (GD)	UAS-*Cyp28d1*	UAS-*Cyp28d2*	UAS-*Ugt86Dd*
30844	0.93 (0.060)	0.94 (0.046)^ns^	0.86 (0.055)*	0.89 (0.083)^ns^
30828	0.90 (0.060)	0.92 (0.061)^ns^	0.88 (0.059)^ns^	0.90 (0.079)^ns^
c42	0.85 (0.074)	0.92 (0.039)*	0.91 (0.066)^ns^	0.94 (0.051)**
uro	0.69 (0.211)	0.94 (0.063)**	0.91 (0.045)**	0.95 (0.023)**
c710	0.74 (0.079)	0.91 (0.068)***	0.88 (0.061)***	0.92 (0.061)***
c724	0.77 (0.083)	0.89 (0.072)**	0.81 (0.077)^ns^	0.93 (0.054)***

We knocked down expression of three genes, using UAS constructs from the VDRC-GD collection, with six different Gal4 drivers. The 30844 driver expresses Gal4 in the hindgut, ureter, and malpighian tubules, 30828 expresses Gal4 in the malpighian tubules, c42-Gal4 and uro-Gal4 are specific to the principal cells of the malpighian tubules, and c710-Gal4 and c724-Gal4 are specific to tubule stellate cells. The phenotype of each genotype was tested across 10 replicate vials, and each gene knockdown was compared to the relevant co-isogenic control using a *t*-test (^ns^ *P* ≥ 0.05, * *P* < 0.05, ** *P* < 0.01, *** *P* < 0.001). The viability of each genotype was also tested two to five times under no-drug conditions. The lowest genotype mean under no-drug conditions was 0.88, and the average genotype mean was 0.95. Notably, the mean phenotypes under no-drug conditions for the non-RNAi, control genotypes were 0.91–0.98, implying that the marked reduction in viability for some of these genotypes on nicotine-supplemented media is not due to general viability defects.

This result—heightened resistance to a drug after knocking down expression of known detoxification genes in cell types known to be involved in xenobiotic metabolism—is counterintuitive. Indeed, RNAi knockdown of the P450 gene *Cyp6g1* in principal cells robustly leads to a reduction in DDT resistance (Yang *et al.* 2007). We speculate that the presence of nicotine or its byproducts, coupled with a reduction in expression of key nicotine metabolizers in the malpighian tubule principal/stellate cells, leads to the production of additional detoxification enzymes, possibly in other tissues, that enhance resistance. Analyses of gene expression in the malpighian tubules and other gut tissues of these knockdown genotypes may provide insight into this observation.

### Overexpression of functional *Ugt86Dd* enhances nicotine resistance

Using the *Ugt86Dd* sequence from the relatively susceptible A3 founder and the relatively resistant A4 founder, we made UAS-*Ugt86Dd* strains, allowing ectopic overexpression of the Ugt86Dd gene product. In the course of verifying clones prior to plasmid injection, we noticed that exon 2 of the A3 allele contained a 22-bp deletion relative to A4, leading to a frameshift and a premature stop codon, reducing the length of the predicted protein product from 517 to 206 amino acids. PCR and sequencing of the original founders revealed that the A3 and A4 strains differ at this variant.

Ubiquitous overexpression of UAS-*Ugt86Dd^A4^* via *Act5C*-Gal4 did not yield viable Gal4-UAS offspring under no-drug conditions, while similar overexpression of UAS-*Ugt86Dd^A3^* did yield Gal4-UAS progeny. These observations imply that ubiquitous overexpression of full-length *Ugt86Dd* is poisonous to cells and confirms that A3 carries a nonfunctional *Ugt86Dd* allele.

**Figure 3:**
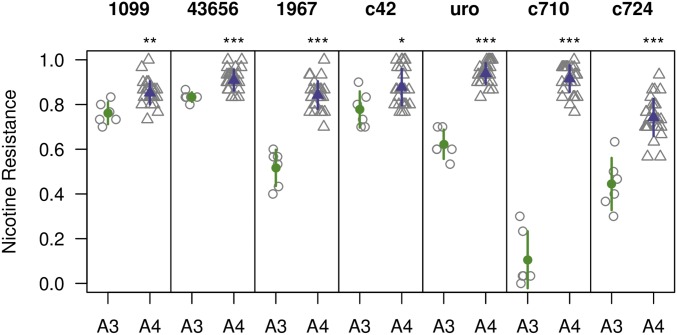
Effect of tissue-specific overexpression of *Ugt86Dd* alleles. We overexpressed *Ugt86Dd* derived from strains A3 (which contains a 22-bp out-of-frame coding deletion) and A4 (which carries an allele making a full-length gene product) using seven tissue-specific Gal4 drivers. We had access to a single UAS-*Ugt86Dd^A3^* transgene line and five UAS-*Ugt86Dd^A4^* transgene lines and tested each line over six replicate nicotine vials (gray open symbols). We present the mean (±1 SD) phenotype of each genotype in colored symbols and compared the genotype means for each Gal4 driver using *t*-tests (* *P* < 0.05, ** *P* < 0.01, *** *P* < 0.001). In every case, the A4 transgene leads to higher nicotine resistance. Notably, we additionally tested all genotypes across two to three no-drug vials, and the A3 transgene showed viabilities of 0.84–0.96. This indicates that the reductions in viability under nicotine conditions are nicotine specific and likely not due to expression of the UAS-*Ugt86Dd^A3^* transgene being generally deleterious.

In order to test the effect of *Ugt86Dd* overexpression on nicotine resistance we used a series of drivers expressing Gal4 in various regions of the gut and compared a single line containing the UAS-*Ugt86Dd^A3^* transgene to five strains containing the same UAS-*Ugt86Dd^A4^* transgene. Figure 3 shows that for every Gal4 driver, overexpression of UAS-*Ugt86Dd^A4^* (containing the insertion allele of *Ugt86Dd*) leads to higher nicotine resistance than overexpression of UAS-*Ugt86Dd^A3^* (containing the deletion). These data suggest that the InDel variant segregating between strains A3 and A4 may be responsible for some of the difference in nicotine resistance exhibited by these strains. We note that while all six strains tested are homozygous for the same third chromosome harboring the transgene landing site, they may be variable on the second and X chromosomes due to postinjection crossing against balancers. Thus, it is conceivable that some of the variation observed between the A3 and A4 transgenes is due to differences in genetic background.

This overexpression experiment may not say anything about the natural site of *Ugt86Dd*-based nicotine detoxification, since we are ectopically expressing the gene at high levels in tissues where it may normally be expressed at more modest levels under its native promoter.

### The *Ugt86Dd* InDel variant is associated with nicotine resistance

Four of the DSPR founder strains (A3, AB8, B6, and B7) possess the 22-bp deletion allele at *Ugt86Dd* (Figure S1). At the Q4 QTL identified by Marriage *et al.* (2014) these four founders have the lowest strain effects, suggesting the InDel may have a functional role in resistance. Furthermore, mean phenotypes of RILs carrying the insertion and deletion alleles are highly significantly different for both the pA and pB populations (*t*-test, *P* < 10^−15^; File S3), with the mean resistance of the deletion-containing RILs being 37% (pA) and 27% (pB) less than that of the insertion-containing RILs.

**Figure 4:**
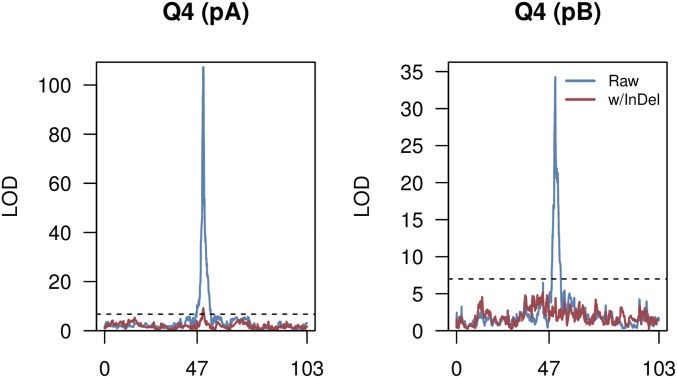
An InDel variant in *Ugt86Dd* explains variation at a nicotine resistance QTL. Each plot shows the LOD curve along chromosome 3, with genetic distance along the *x*-axis, in the pA (left) and pB (right) DSPR populations. In blue, using a standard mapping analysis in the DSPR, one sees evidence for the major-effect QTL Q4. In red, using a similar analysis but now including a covariate accounting for the InDel status of each phenotyped RIL, one sees a peak in pA with a LOD score just surviving the threshold, and no peak in pB. These data suggest the InDel variant contributes to the effect observed at Q4.

We employed InDel status as an additional covariate in the DSPR QTL mapping analysis and observed a substantial reduction in LOD score at the site of the Q4 locus (Figure 4), eliminating any above-threshold peak in pB and leaving a much more modest effect at the locus in pA; in the standard analysis, Q4 contributes 46.5% to the variation among pA lines, whereas the above-threshold peak at the same location after accounting for the InDel contributes 5.3% to the variation. These data imply that the InDel variant alone, or one or a collection of variants in linkage disequilibrium (LD) with this variant, are responsible for a large fraction of the nicotine resistance variation in the DSPR and may completely, or nearly completely, explain the major-effect QTL mapped by Marriage *et al.* (2014).

To attempt to validate the effect of the InDel variant on nicotine resistance, we exploited the DGRP, a collection of ∼200 inbred, resequenced strains of *D. melanogaster* (Mackay *et al.* 2012; Huang *et al.* 2014). Seven of the DGRP strains were found to harbor the same 22-bp deletion allele as found in the DSPR. We intercrossed these lines for seven generations to generate a population fixed for the deletion allele, but otherwise outbred, and generated a similar population from seven random DGRP strains carrying the insertion allele. Comparing these populations revealed a small, yet significant reduction in resistance in the deletion population (insertion population mean = 0.94, deletion population mean = 0.89, *t*-test, *P* = 0.011). While this result does tend to validate our work in the DSPR, any effect of the *Ugt86Dd* InDel variant is apparently much smaller in the DGRP.

### CRISPR/Cas9-derived deletion mutations at *Ugt86Dd* in A4 reduce resistance

The effect of the deletion allele in founder A3 is to generate a premature stop codon in *Ugt86Dd*. To directly test the effect of premature stop-encoding mutations in this gene, we generated a series of deletion alleles via CRISPR/Cas9 editing. We constructed a custom injection strain containing an X-linked *vasa*-Cas9 transgene and an A4-derived third chromosome and employed a gRNA that generates double-strand breaks at the site of InDel (Figure S1). We generated a series of 16 independent mutations (File S4), 13 of which lead to premature stop codons, and constructed strains carrying homozygous edited third chromosomes for all 16. Simultaneously, we made seven strains with homozygous unedited third chromosomes from genotypes that were passed through the CRISPR/Cas9 process (injection, balancing, and so on). Our rationale for keeping such genotypes was that during the creation of our injection strain, and while establishing homozygous edited chromosomes, we employed several generations of crossing with balancers. Since movement of genetic material via gene conversion from a balancer to the nonbalancer homolog has been inferred (Blumenstiel *et al.* 2009), such unedited genotypes provided valuable controls for the effects of induced mutations. All 23 strains were tested in multiple replicates for viability under no-drug and nicotine conditions, and while we observed no difference between the sets of unedited and edited genotypes in the absence of nicotine (*P* = 0.2), we saw a large decrease in resistance due to editing (*P* < 10^−15^, Table S1).

The fact that the three lesions that result in amino acid changes, but do not introduce a premature stop codon, still each lead to a strong reduction in nicotine resistance may suggest that the region of the protein targeted for mutation is critical to the function of Ugt86Dd.

**Figure 5:**
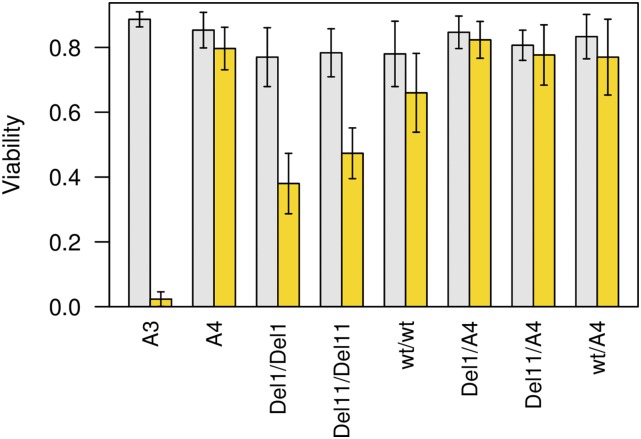
Phenotypes of background-controlled *Ugt86Dd* CRISPR/Cas9-derived mutations. We made a pair of *Ugt86Dd* mutations homozygous in the A4 background. *Del1* introduces a 1-bp mutation, while *Del11* introduces an 11-bp mutation, each leading to a different stop codon mutation (File S4). In addition, we passed an unedited allele (*wt*) through an identical crossing scheme, which should be identical to the pure A4 strain. We tested 10 replicate vials of each of the genotypes in the plot for both no-drug (gray bars) and nicotine (yellow bars) treatments and present the mean (±1 SD) viability across replicate vials. The two homozygous mutant strains (*Del1*/*Del1* and *Del11*/*Del11*) have significantly lower nicotine resistance (*t*-test, *P* < 10^−5^) in comparison to the parental A4 genotype and the unedited control strain (*wt*/*wt*). Since the unedited control strain has slightly, but significantly reduced resistance in comparison to A4 (*P* < 0.01), the crossing scheme used to establish the homozygous CRISPR/Cas9-derived genotypes may have resulted in some additional changes in addition to the target mutation.

For two deletion alleles—a 1-bp and an 11-bp deletion—that both lead to premature stop codons, along with one unedited allele, we used standard fly genetics to put the third chromosome (originally derived from A4) into a complete A4 background, generating strains A4-*Ugt86Dd^Del1^*, A4-*Ugt86Dd^Del11^*, and A4-*Ugt86Dd^wt^*. All three have marginally reduced viability under no-drug conditions compared to the original A4 founder strain (*P* = 0.03–0.06), perhaps indicative of novel mutations relative to the A4 progenitor line, or slight changes in sequence due to gene conversion from balancers during stock construction. Nonetheless, Figure 5 shows that both of the deletion-carrying stocks A4-*Ugt86Dd^Del1^* and A4-*Ugt86Dd^Del11^* have significantly reduced nicotine resistance in comparison with A4-*Ugt86Dd^wt^* (*P* < 10^−4^ and *P* < 0.001, respectively). The induced mutations appear to behave recessively, since measuring the phenotype of their heterozygous progeny after crossing to A4 recapitulates the A4 homozygous phenotype (Figure 5).

The difference in nicotine resistance between strains A3 and A4 is 0.69–0.77 (Figure 1 and Figure 5), while A4-*Ugt86Dd^Del1^* and A4-*Ugt86Dd^Del11^* differ from A4-*Ugt86Dd^wt^* by 0.28 and 0.19, respectively. This suggests that 25–41% of the difference between founders A3 and A4 could be due to functional variation at *Ugt86Dd*, with the most likely variant conferring this effect being the naturally segregating 22-bp InDel. Since the difference between the mean phenotype of RILs carrying the A3 and A4 alleles at the Q4 locus is 0.37 (Marriage *et al.* 2014), our gene editing data again imply that *Ugt86Dd*, and likely the InDel variant that results in an allele containing a similar premature stop codon to the edited mutations, confers the bulk of the effect observed at this QTL.

## Discussion

### Variation at *Cyp28d1* may underlie nicotine resistance

Quantitative complementation tests suggest that functional allelic variation is present at *Cyp28d1* (Figure S2). Previous RNAseq data (Marriage *et al.* 2014) suggest gene expression is higher in the more resistant A4 line than in the more susceptible A3 line, and both ubiquitous and anterior midgut-specific RNAi of *Cyp28d1* led to a reduction in nicotine resistance (Figure 2 and Table 1). These results strongly implicate variation at *Cyp28d1* in the genetic control of nicotine resistance. Data from Chakraborty *et al.* (2017), who have generated an assembly of DSPR founder A4 based on long-read sequencing data, give some indication that variation in *Cyp28d1* copy number may be causative; in contrast to the *D. melanogaster* reference strain, founder A4 possesses two copies of *Cyp28d1*. The relatively high expression of *Cyp28d1* in A4 (Marriage *et al.* 2014) supports the idea of a positive correlation between copy number and gene expression, which could in turn lead to enhanced resistance. Such positive associations between P450 copy number and xenobiotic resistance have been reported previously (Wondji *et al.* 2009; Schmidt *et al.* 2010). Nonetheless, the relationship between copy number and expression level is not straightforward (Zhou *et al.* 2011; Schrider *et al.* 2016), and while the presence of two copies of *Cyp28d1* in a strain resistant to nicotine is suggestive, establishing a direct effect requires functional validation.

Evidence that *Cyp28d2* also contributes to nicotine resistance, and segregates for causative variation generating the Q1 locus, is marginally more mixed. A quantitative complementation test using an insertional mutant implies the gene harbors a functional difference between strains A3 and A4 (Figure S2), strain A4 exhibits higher expression than A3 (Marriage *et al.* 2014), and ubiquitous knockdown of *Cyp28d2* decreases resistance (Figure 2). However, knockdown of the gene in the midgut had no detectable effect (Table 1). It is possible that we did not knock down *Cyp28d2* in the tissue in which it would normally act on nicotine, and failed to identify an effect. Employing an expanded catalog of Gal4 drivers (Buchon *et al.* 2013) may uncover the tissue in which *Cyp28d2* is active against nicotine. Given our data, either *Cyp28d1* or *Cyp28d2*, or indeed both genes, may contribute to the effect observed at the Q1 locus originally mapped by Marriage *et al.* (2014). Additional fine mapping in natural populations, where both genes appear to segregate for copy number variants (CNVs) (Good *et al.* 2014), may be profitable and allow functional effects associated with the two genes to be separated.

### *Ugt86Dd* explains a large fraction of the variation for nicotine resistance in the DSPR

Deficiency quantitative complementation tests suggest that the set of UGT genes under Q4 may harbor functional allelic variation affecting nicotine resistance, although these tests do not directly implicate *Ugt86Dd* (Figure S2). RNAseq, both from the present study and Marriage *et al.* (2014), suggest *Ugt86Dd* is more highly expressed in more resistant genotypes. Knocking down the gene ubiquitously (Figure 2) and in the anterior midgut (Table 1) leads to reduced resistance, while overexpression of full-length *Ugt86Dd* enhances resistance compared to overexpression of a putatively null allele (Figure 3). All this evidence strongly implicates *Ugt86Dd* as a major source of genetic variation contributing to nicotine resistance.

The segregating 22-bp coding InDel is a compelling candidate for the actual causative site underlying much of this variation. By accounting for the polymorphism during QTL mapping the effect of the Q4 interval is markedly reduced (Figure 4), and comparison of DGRP-derived populations fixed for alternate alleles at the InDel suggest the deletion is associated with reduced resistance. Finally, while we do not specifically generate the 22-bp deletion in the A4 background, our creation and testing of other deletions at a similar location in the *Ugt86Dd* gene, which similarly lead to premature stop codons, provides strong evidence that the InDel is functional, and directly leads to an effect on nicotine resistance (Figure 5).

Several pieces of evidence suggest that additional polymorphisms also contribute to the effect observed at the Q4 locus. First, the strain effects at the mapped QTL do not fall into two categories, as would be expected if a biallelic causative polymorphism was solely responsible for the QTL effect (Marriage *et al.* 2014). Second, a quantitative complementation test using a deficiency that eliminates several UGT genes, but leaves *Ugt86Dd* intact, showed a significant failure to complement, implying other genes in the QTL region may segregate for functional variation (Figure S2). Third, after accounting for the InDel, QTL mapping in the pA population still yields a modest-effect QTL at the Q4 interval, suggesting factors not in perfect LD with the InDel in the DSPR are additionally involved. Such variants could impact other genes in the Q4 interval, but potentially might affect the regulation and/or coding sequence of *Ugt86Dd* in alleles derived from founders other than A3 and A4. Finally, the difference between the strain effects of A4 (which carries the insertion at *Ugt86Dd*) and A3 (which carries the deletion) at QTL Q4 is larger than the phenotypic difference between wild-type and edited mutant *Ugt86Dd* alleles in the same A4 background. Assuming the effects of the naturally occurring deletion at *Ugt86Dd* in A3 and the CRISPR/Cas9-induced deletion in A4 are the same, this observation is most likely due to variants in other genes in the QTL interval.

One factor that could contribute to the Q4 QTL in addition to *Ugt86Dd* is a CNV at *Ugt86Dh* (Chakraborty *et al.* 2017), where founder A4 harbors two copies of this gene relative to the *D. melanogaster* reference genome (although we do not yet know the status of this gene in other DSPR founders.) Notably two copies of *Ugt86Dh* increase expression (Chakraborty *et al.* 2017), implying the CNV may have some functional effect, and the gene is deleted in the one deficiency that does not eliminate *Ugt86Dd* (Figure S2). Given the circumstantial evidence associated with *Ugt86Dh*, it is a strong candidate for additional follow-up functional characterization.

### Effect of the segregating InDel at *Ugt86Dd*

Given the apparent large effect of the InDel in the DSPR, and specifically the effect of introducing a premature stop-encoding lesion into A4, we anticipated the effect of the deletion would routinely be large. However, comparison of DGRP-derived populations fixed for alternate alleles at the InDel showed only a modest effect of the variant. Our design assumes we can estimate the effect of each allele at the InDel averaged over a similarly randomized genetic background. However, due to the paucity of DGRP lines possessing the deletion, it is likely that the insertion and deletion populations show many other fixed differences along the genome, some of which could lead to underestimating any true effect of the variant.

Outside of this technical challenge, one possibility for the very different effect estimates of the InDel in DSPR- and DGRP-derived populations is the different genetic background of the two panels. The DSPR is derived from a worldwide collection of *P*-element-free strains that have been present in laboratories for decades (King *et al.* 2012b), while the DGRP is a set of strains relatively recently collected from North Carolina (Mackay *et al.* 2012). One observation suggesting these populations may have different genetic architectures for nicotine resistance comes from work by Passador-Gurgel *et al.* (2007). This study demonstrated that flies derived from North Carolina are commonly more nicotine resistant than flies derived from California. Indeed, using our assay we find that average nicotine resistance in our DGRP-derived outbred populations is higher than all but three of the 1274 DSPR RILs assayed by Marriage *et al.* (2014). While we can only speculate that there is an interaction between the InDel and variation at other functional variants, we know that epistasis contributes significantly to phenotypic variation (Monnahan and Kelly 2015; Forsberg *et al.* 2017), and that large-effect mutations moved into a varied set of genetic backgrounds result in a spectrum of phenotypic expression (Chandler *et al.* 2013; Chari and Dworkin 2013; He *et al.* 2014; Sittig *et al.* 2016). Thus, there is the potential for genetic background to influence the phenotypic expression of the *Ugt86Dd* variant.

A way to both establish the causative effect of the 22-bp *Ugt86Dd* InDel in the DSPR, as well as to estimate any background-specific expression of the variant alleles in the DGRP (and other populations), is to use CRISPR/Cas9 to precisely introduce the deletion into strains carrying a wild-type *Ugt86Dd* sequence, and similarly “repair” the allele in strains carrying the deletion (see Gratz *et al.* 2013). Such reagents would additionally facilitate future work on the mechanisms underlying nicotine detoxification by *Ugt86Dd*, for instance, by examining the products of nicotine metabolism, and allow tests of the effect of the *Ugt86Dd* gene in the detoxification of other xenobiotics, perhaps neonicotinoid insecticides.

### *Ugt86Dd* deletion is rare and recessive

Among the 15 DSPR founder strains, the 22-bp deletion allele at *Ugt86Dd* is fairly common (4/15 or 27%). To assess its frequency in wild-caught population samples, we searched for next-generation sequencing reads that perfectly match the deletion and insertion alleles at the variant in a series of pooled resequencing data sets derived from a number of United States populations (see *Materials and Methods* and Bergland *et al.* 2014). Averaging over these samples we roughly estimate the frequency of the deletion in nature at ∼2% (Table S2), although there is fairly large sample-to-sample variation around this value (0–11%) that could represent real biological, among-population variation, or simply be a result of sampling (of both individuals and sequencing reads.)

The deletion allele leads to a premature stop, likely ablating gene function, and purifying selection may perhaps explain the low frequency of the allele in nature. Huang *et al.* (2014) identified variants in the set of 200 DGRP fly strains that potentially generate a damaged protein, and found that such variants are typically less frequent than other sites (*e.g.*, nonsynonymous variants). Similarly, studies in humans have shown that rare, protein-coding polymorphisms are enriched for putatively damaging events (MacArthur *et al.* 2012; Nelson *et al.* 2012; Tennessen *et al.* 2012). Since the *Ugt86Dd* deletion appears to be recessive (Figure 5), under the assumption there is some cost to an organism in nature carrying the deletion in homozygous form, this might explain why its frequency in nature is not even lower.

It is not clear why the deletion allele is fairly common in the DSPR founders. The founders were collected in the 1950s and 1960s from a number of different countries (King *et al.* 2012b), prior to the *P*-element sweep through *D. melanogaster*, and conceivably populations were enriched for the deletion during this period. However, given the small number of founders, and their arbitrary sampling from stock centers many generations after their original derivation from nature (King *et al.* 2012b), a deletion frequency of 4/15 may not reflect the actual worldwide population frequency of the allele at the time of collection. It is tempting to speculate that the frequency of the *Ugt86Dd* deletion allele has been modulated by the use of nicotine as an insecticide. Nicotine was in common use as a pesticide after World War II (Shepard 1951; Metcalf 1955), but is no longer used in this way in the United States (Environmental Protection Agency 2009), having been supplanted by other insecticides, many of which are nicotine derivatives (Goulson 2013). However, it is known that individual UGT genes can act on a range of substrates (Tukey and Strassburg 2000), so any selection acting on *Ugt86Dd* could be due to one or more unknown compounds to which flies can be exposed in nature.

Despite the large effect of the InDel in the DSPR, replicated by our CRISPR/Cas9 editing, the recessive nature of the deletion allele and its low frequency in nature likely means the InDel explains only a very small fraction of the variation in nicotine resistance in a natural, outbred diploid population. Using the formula appropriate for a completely recessive variant, *V*_a_ = 8*pq*^3^*α*^2^ (Falconer and Mackay 1996), estimating the frequency (*q*) of the deletion allele at 2%, taking the effect of the deletion from the difference between the background matched wild-type *Ugt86Dd* genotype and the CRISPR-derived lesions (0.19–0.28), and assuming the phenotypic variance among DSPR line means (Marriage *et al.* 2014) is representative of that in a natural population, the InDel explains <0.01% of the phenotypic variation for the trait. This estimate is lower still if the difference between homozygous insertion- and deletion-carrying genotypes are more in line with the values observed for the DGRP populations.

### Resolving causative polymorphisms underlying QTL

Several features of our experimental system facilitated the resolution of a likely causative sequence variant underlying a mapped QTL. First, our study of a xenobiotic resistance trait clearly motivated a focus on the small number of detoxification family members within QTL. This ability to home in on genes that were *a priori* highly likely to harbor segregating causative variation may be unavailable for many other traits, where the purpose of unbiased, genomewide mapping is to help elucidate the pathways underlying variation. Second, we were fortunate that the causative gene possessed a variant with a clear molecular signature of damage. The phenotypic effects of most polymorphisms, particularly those associated with noncoding changes, are considerably more challenging to decipher from sequence data alone. Third, despite the QTL containing multiple detoxification genes, and our prediction that the QTL was generated by the action of multiple genes/alleles (Marriage *et al.* 2014), the *Ugt86Dd* coding mutation alone has a major effect on phenotype that facilitated its detection. Other study systems will lack these advantages. Despite these factors, all studies in *Drosophila* can leverage the battery of functional tools available for this organism to drill down to the causative molecular lesion(s) underlying QTL and gain insight into the genetic control of trait variation.

## Supplementary Material

Supplemental material is available online at www.genetics.org/lookup/suppl/doi:10.1534/genetics.117.300058/-/DC1.
