# Supplementary material for: Naturally Segregating Variation at Ugt86Dd Contributes to Nicotine Resistance in Drosophila melanogaster
Source: Genetics. 2017 Jul 26;207(1):311–25. doi: 10.1534/genetics.117.300058 (PMC5586381; doi:10.1534/genetics.117.300058)
Supplement: Supplementary file 1 [file 311FigureS1.pdf]

Figure S1 Sequence of the *Ugt86Dd* 22-bp InDel region in the 15 DSPR founder strains. The stretch of DNA is within coding exon 2 of the *Ugt86Dd* gene, and the genome coordinates are relative to Release 6 of the *Drosophila melanogaster* reference. SNPs in red are synonymous. The 22-bp deletion in founders A3, B6, B7, and AB8 leads to a short incorrect amino acid sequence (blue) followed by a premature stop codon. The sequence highlighted in yellow is the guide RNA sequence used to generate double-strand breaks ("DSB") via CRISPR/Cas9 editing in A4.

```
     3R:11,127,692                        DSB                                    3R:11,127,600
                          TCACTACGAAGTCATTGTGGAGG

A1   ACCTTTCTGGAGCGCTTGGAAAATCACTACGAAGTCATTGTGGAGGACATTCATCGCCAATTCGTTCACCTGCCACATATGAGAAATGTTTAC
A2   ACCTTTCTGGAGCGCTTGGAAAATCACTACGAAGTCATTGTGGAGGACATTCATCGCCAATTCGTTCACCTGCCACATATGAGAAATGTTTAC
A4   ACGTTTCTGGAGCGCTTGGAAAATCACTACGAAGTCATTGTGGAGGACATTCATCGCCAATTCGTTCACCTGCCACATATGAGAAATGTTTAC
A5   ACCTTTCTGGAGCGCTTGGAAAATCACTACGAAGTCATTGTGGAGGACATTCATCGCCAATTCGTTCACCTGCCACATATGAGAAATGTTTAC
A6   ACGTTTCTGGAGCGCTTGGAAAATCACTACGAAGTCATTGTGGAGGACATTCATCGCCAATTCGTTCACCTGCCACATATGAGAAATGTTTAC
A7   ACGTTTCTGGAGCGCTTGGAAAATCACTACGAAGTCATTGTGGAGGACATTCATCGCCAATTCGTTCACCTGCCACATATGAGAAATGTTTAC
B1   ACGTTTCTGGAGCGCTTGGAAAATCACTACGAAGTCATTGTGGAGGACATTCATCGCCAATTCGTTCACCTGCCACATATGAGAAATGTTTAC
B2   ACCTTTCTGGAGCGCTTGGAAAATCACTACGAAGTCATTGTGGAGGACATTCATCGCCAATTCGTTCACCTGCCACATATGAGAAATGTTTAC
B3   ACCTTTCTGGAGCGCTTGGAAAATCACTACGAAGTCATTGTGGAGGACATTCATCGCCAATTCGTTCACCTGCCACATATGAGAAATGTTTAC
B4   ACGTTTCTGGAGCGCTTGGAAAATCACTACGAAGTCATTGTGGAGGACATTCATCGCCAATTCGTTCACCTGCCACATATGAGAAATGTTTAC
B5   ACCTTTCTGGAGCGCTTGGAAAATCACTACGAAGTCATTGTGGAGGACATTCATCGCCAATTCGTTCACCTGCCACATATGAGAAATGTTTAC
       T  F  L  E  R  L  E  N  H  Y  E  V  I  V  E  D  I  H  R  H  F  V  H  L  P  H  M  R  N  V  Y

A3   ACCTTTCTGGAGCGCTTGGAAAATCACTACGAAGT----------------------GAATTCGTTCACCTGCCACATATGAGAAATGTTTAC
B6   ACCTTTCTGGAGCGCTTGGAAAATCACTACGAAGT----------------------GAATTCGTTCACCTGCCACATATGAGAAATGTTTAC
B7   ACCTTTCTGGAGCGCTTGGAAAATCACTACGAAGT----------------------GAATTCGTTCACCTGCCACATATGAGAAATGTTTAC
AB8  ACCTTTCTGGAGCGCTTGGAAAATCACTACGAAGT----------------------GAATTCGTTCACCTGCCACATATGAGAAATGTTTAC
       T  F  L  E  R  L  E  N  H  Y  E                        V  N  S  F  T  C  H  I  X
```
