# Supplementary material for: Naturally Segregating Variation at Ugt86Dd Contributes to Nicotine Resistance in Drosophila melanogaster
Source: Genetics. 2017 Jul 26;207(1):311–25. doi: 10.1534/genetics.117.300058 (PMC5586381; doi:10.1534/genetics.117.300058)

Figure S2 Results of quantitative complementation tests. We carried out tests using a series of deficiencies and insertional mutants. The "Interaction" *P*-value presented assesses the significance of the Founder × Mutant interaction, determining whether there is a significant quantitative failure to complement. Information regarding the positions of deficiencies/insertions is reported based on Release 6 of the *Drosophila melanogaster* reference genome. Information regarding the genes deleted was taken from FlyBase on March 11, 2017.

<u>2L Deficiencies</u>

BDSC: 7497
Exelixis Deficiency
w^{1118}; Df(2L)Exel6011/CyO

Deletes: 2L:5,147,258..5,305,646
13 protein-coding genes including: *Cyp28d1*, *Cyp28d2*, *Cyp4ac1*, *Cyp4ac2*, and *Cyp4ac3*

Interaction: $P < 10^{-9}$

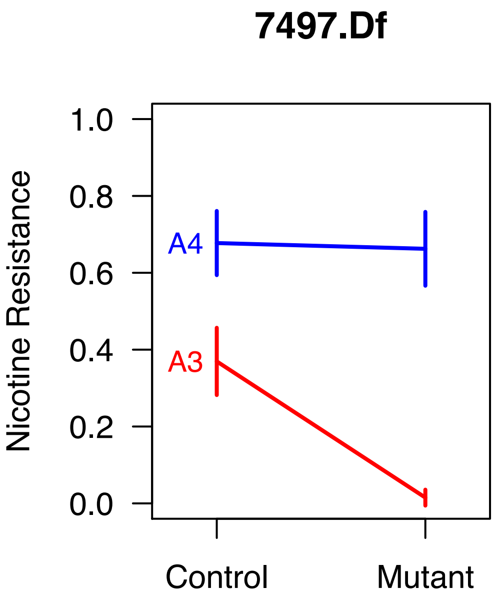

BDSC: 26545
BSC Deficiency
w^{1118}; Df(2L)BSC693/SM6a

Deletes: 2L:5,209,495..5,305,646
11 protein-coding genes including: *Cyp28d1*, *Cyp4ac1*, *Cyp4ac2*, and *Cyp4ac3*

Interaction: $P < 10^{-10}$

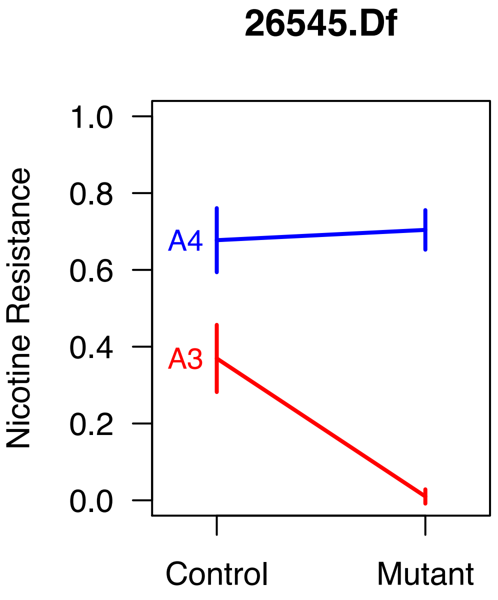

3R Deficiencies

BDSC: 7957
Exelixis Deficiency
w^{1118}; Df(3R)Exel7306/TM6B, Tb^{1}

Deletes: 3R:10,871,007..11,156,829
18 protein-coding genes including:
Ugt86Dd, *Ugt86Di*, and *Ugt86Dc*

Interaction: $P < 10^{-8}$

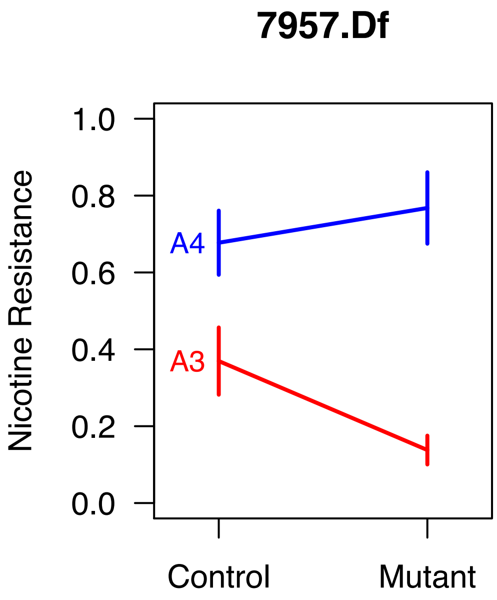

BDSC: 7958
Exelixis Deficiency
w^{1118}; Df(3R)Exel8152/TM6B, Tb^{1}

Deletes: 3R:11,154,150..11,200,280
11 protein-coding genes including:
Ugt86Dc, *Ugt86Da*, *Ugt86Dg*,
Ugt86De, *Ugt35b*, *Ugt35a*,
Ugt86Dj, and *Ugt86Dh*

Interaction: $P < 10^{-6}$

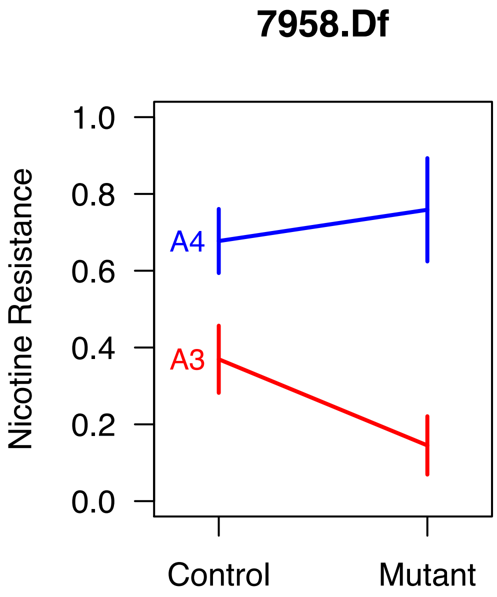

BDSC: 9083
DrosDel Deficiency
w^{1118}; Df(3R)ED5506/TM6C, cu^{1} Sb^{1}

Deletes: 3R:10,884,998..11,172,748
19 protein-coding genes including:
Ugt86Dd, *Ugt86Di*, *Ugt86Dc*,
Ugt86Da, *Ugt86Dg*, *Ugt86De*,
Ugt35b, and *Ugt35a*

Interaction: $P < 10^{-9}$

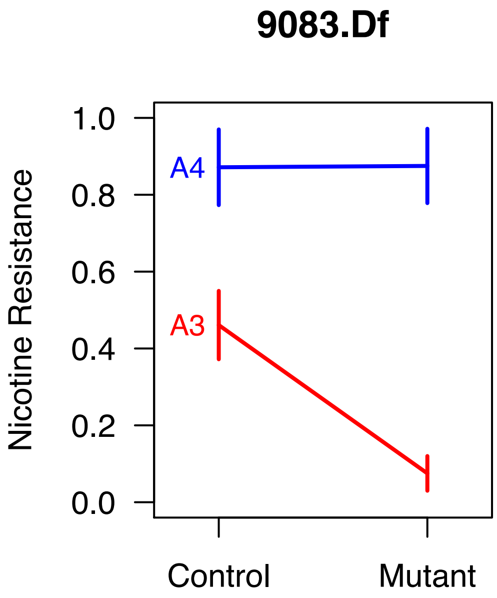

Minos Insertion Mutants (2L)

BDSC: 23530
Minos Insertion
w^{1118}; Mi{ET1}Cyp28d1^{MB03293}

Insertion: 2L:5,211,244
Inserts within *Cyp28d1* coding exon

Interaction: $P < 10^{-5}$

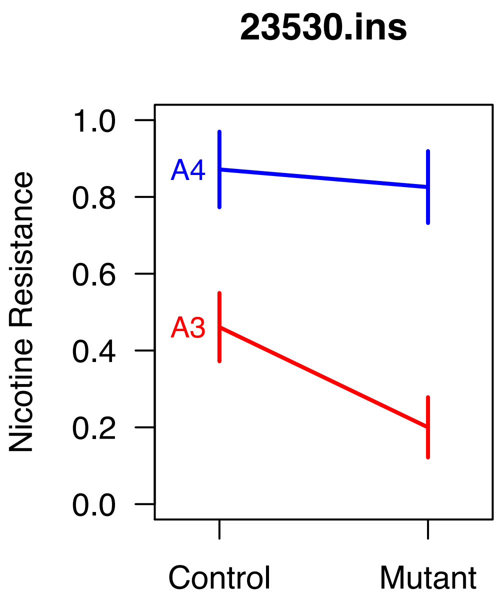

BDSC: 23587
Minos Insertion
w^{1118}; Mi{ET1}Cyp28d2^{MB02776}

Insertion: 2L:5,208,263
Inserts within *Cyp28d2* coding exon

Interaction: $P < 10^{-14}$

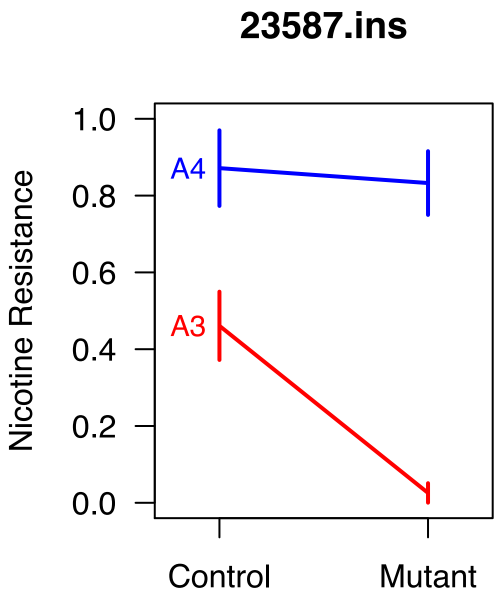

Minos Insertion Mutants (3R)

BDSC: 24834
Minos Insertion
w^{1118}; Mi{ET1}Ugt86Dj^{MB04890}

Insertion: 3R:11,173,796
Inserts within *Ugt86Dj* coding exon

Interaction: $P < 0.001$

Note that there is a larger difference between founder alleles in the 'Control' background than in the 'Mutant' background. This observation is not consistent with an allelic failure to complement, and more likely indicates epistasis.

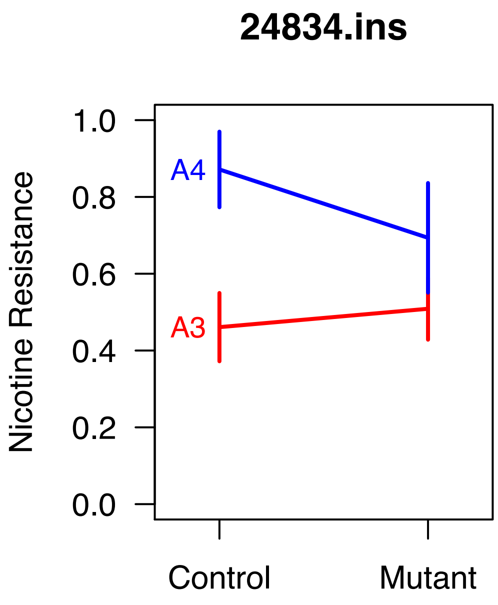

BDSC: 27861
Minos Insertion
w^{1118}; Mi{ET1}Ugt86Dh^{MB11311}

Insertion: 3R:11,177,673
Inserts within the 3'UTR of one of the two *Ugt86Dh* isoforms

Interaction: $P < 0.01$

Note that there is a larger difference between founder alleles in the 'Control' background than in the 'Mutant' background. This observation is not consistent with an allelic failure to complement, and more likely indicates epistasis.

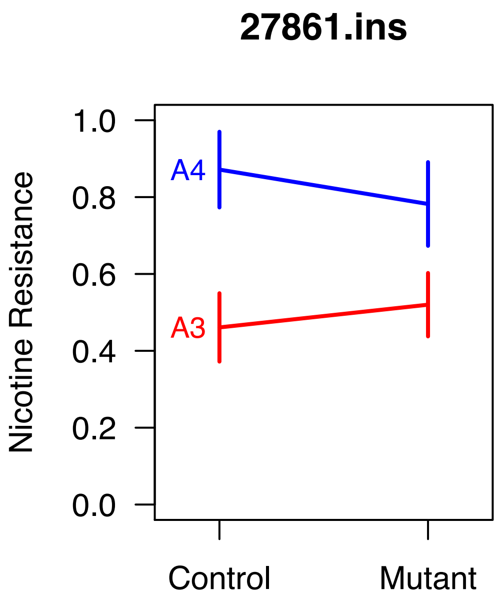

Supplement: Supplementary file 2 [file 311FigureS2.pdf]
