# Supplementary material for: Naturally Segregating Variation at Ugt86Dd Contributes to Nicotine Resistance in Drosophila melanogaster
Source: Genetics. 2017 Jul 26;207(1):311–25. doi: 10.1534/genetics.117.300058 (PMC5586381; doi:10.1534/genetics.117.300058)
Supplement: Supplementary file 4 [file 311FileS2.pdf]

File S2 The set of pB2 DSPR RILs used for RNAseq. RIL IDs are given as the standard DSPR nomenclature (see FlyRILs.org). The nicotine resistance phenotype data is taken directly from that presented in Marriage et al. (2014). The allele present in each RIL at the *Ugt86Dd* 22-bp InDel (In = insertion, Del = 22-bp deletion) is based on direct genotyping of the DSPR founder strains, and high confidence estimates of the founder contribution at the *Ugt86Dd* gene region in each RIL.

High resistance RILs (*n* = 6)

RIL ID	Pheno	InDel Allele
22098	0.80	In
22110	0.87	In
22112	0.83	In
22156	0.83	In
22290	0.77	Del
22342	0.77	In

Low resistance RILs (*n* = 12)

RIL ID	Pheno	InDel Allele
22045	0.00	Del
22097	0.00	Del
22124	0.00	Del
22127	0.00	Del
22128	0.00	Del
22163	0.00	Del
22226	0.00	Del
22232	0.00	Del
22281	0.00	Del
22301	0.00	Del
22317	0.00	Del
22322	0.00	Del
