# Supplementary material for: Naturally Segregating Variation at Ugt86Dd Contributes to Nicotine Resistance in Drosophila melanogaster
Source: Genetics. 2017 Jul 26;207(1):311–25. doi: 10.1534/genetics.117.300058 (PMC5586381; doi:10.1534/genetics.117.300058)
Supplement: Supplementary file 6 [file 311FileS4.pdf]

File S4 Sequences of all CRISPR/Cas9-generated mutations in *Ugt86Dd*. For each of the 16 mutations we give the DNA sequence, and the anticipated resulting amino acid sequence. The genotype IDs match those provided in the remainder of the study.

Wildtype *Ugt86Dd* sequence

```
CGAACCGATCGGATGACCTTTCTGGAGCGCTTGGAAAATCACTACGAAGTCATTGTGGAGGACATTCATCGCCATTTC
 R  T  D  R  M  T  F  L  E  R  L  E  N  H  Y  E  V  I  V  E  D  I  H  R  H  F
GTTCACCTGCCACATATGAGAAATGTTTACAAAAAGTATTTCCCGAATGCAAAGAAAACCCTGGAGGAAGTCATGGAT
 V  H  L  P  H  M  R  N  V  Y  K  K  Y  F  P  N  A  K  K  T  L  E  E  V  M  D
```

CRISPR/Cas9 mutations leading to a premature stop codon

Genotype ID = PremStop[3F.4F]
Genotype ID (following chromosome substitution) = A4-*Ugt86Dd*[Del1]
Mutation = 1bp (T) deleted

```
CGAACCGATCGGATGACCTTTCTGGAGCGCTTGGAAAATCACTACGAAGTCATTGGGAGGACATTCATCGCCATTTCG
 R  T  D  R  M  T  F  L  E  R  L  E  N  H  Y  E  V  I  G  R  T  F  I  A  I  S
TTCACCTGCCACATATGA
 F  T  C  H  I  X
```

Genotype ID = PremStop[8F.3M]
Mutation = 1bp (G) deleted

```
CGAACCGATCGGATGACCTTTCTGGAGCGCTTGGAAAATCACTACGAAGTCATTTGGAGGACATTCATCGCCATTTCG
 R  T  D  R  M  T  F  L  E  R  L  E  N  H  Y  E  V  I  T  R  T  F  I  A  I  S
TTCACCTGCCACATATGA
 F  T  C  H  I  X
```

Genotype ID = PremStop[1M.2F]
Mutation = 2bp (TG) deleted

```
CGAACCGATCGGATGACCTTTCTGGAGCGCTTGGAAAATCACTACGAAGTCATTGGAGGACATTCATCGCCATTTCGT
 R  T  D  R  M  T  F  L  E  R  L  E  N  H  Y  E  V  I  G  G  H  S  S  P  F  R
TCACCTGCCACATATGAGAAATGTTTACAAAAAGTATTTCCCGAATGCAAAGAAAACCCTGGAGGAAGTCATGGATAG
 S  P  A  T  T  E  K  C  L  Q  K  V  F  P  E  C  K  E  N  P  G  G  S  H  G  X
```

Genotype ID = PremStop[7F.4F]
Mutation = 4bp (ATTG) deleted

```
CGAACCGATCGGATGACCTTTCTGGAGCGCTTGGAAAATCACTACGAAGTCTGGAGGACATTCATCGCCATTTCGTTC
 R  T  D  R  M  T  F  L  E  R  L  E  N  H  Y  E  V  T  R  T  F  I  A  I  S  F
ACCTGCCACATATGA
 T  C  H  I  X
```

Genotype ID = PremStop[13M.5F]
Mutation = 4bp (TCAT) deleted

```
CGAACCGATCGGATGACCTTTCTGGAGCGCTTGGAAAATCACTACGAAGTGTGGAGGACATTCATCGCCATTTCGTTC
 R  T  D  R  M  T  F  L  E  R  L  E  N  H  Y  E  V  W  R  T  F  I  A  I  S  F
ACCTGCCACATATGA
 T  C  H  I  X
```

Genotype ID = PremStop[19F.4F]

Mutation = 5bp (GTGGA) deleted

```
CGAACCGATCGGATGACCTTTCTGGAGCGCTTGGAAAATCACTACGAAGTCATTGGACATTCATCGCCATTTCGTTCA
  R  T  D  R  M  T  F  L  E  R  L  E  N  H  Y  E  V  I  G  H  S  S  P  F  R  S
CCTGCCACATATGAGAAATGTTTACAAAAAGTATTTCCCGAATGCAAAGAAAACCCTGGAGGAAGTCATGGATAG
  P  A  T  T  E  K  C  L  Q  K  V  F  P  E  C  K  E  N  P  G  G  S  H  G  X
```

Genotype ID = PremStop[8F.2F]

Mutation = 8bp (GTGGAGGA) deleted

```
CGAACCGATCGGATGACCTTTCTGGAGCGCTTGGAAAATCACTACGAAGTCATTCATTCATCGCCATTTCGTTCACCT
  R  T  D  R  M  T  F  L  E  R  L  E  N  H  Y  E  V  I  H  S  S  P  F  R  S  P
GCCACATATGAGAAATGTTTACAAAAAGTATTTCCCGAATGCAAAGAAAACCCTGGAGGAAGTCATGGATAG
  A  T  T  E  K  C  L  Q  K  V  F  P  E  C  K  E  N  P  G  G  S  H  G  X
```

Genotype ID = PremStop[13M.2M]

Genotype ID (following chromosome substitution) = A4-*Ugt86Dd*^{Del11}

Mutation = 11bp (AGTCATTGTGG) deleted

```
CGAACCGATCGGATGACCTTTCTGGAGCGCTTGGAAAATCACTACGAAGGACATTCATCGCCATTTCGTTCACCTGCC
  R  T  D  R  M  T  F  L  E  R  L  E  N  H  Y  E  G  L  S  S  P  F  R  S  P  A
ACATATGAGAAATGTTTACAAAAAGTATTTCCCGAATGCAAAGAAAACCCTGGAGGAAGTCATGGATAG
  T  T  E  K  C  L  Q  K  V  F  P  E  C  K  E  N  P  G  G  S  H  G  X
```

Genotype ID = PremStop[6M.4F]

Mutation = 15bp (ACGAAGTCATTGTGG) deleted

```
CGAACCGATCGGATGACCTTTCTGGAGCGCTTGGAAAATCACTAG
  R  T  D  R  M  T  F  L  E  R  L  E  N  H  X
```

Genotype ID = PremStop[1F.2F]

Mutation = Overall 1bp insert (G->CA)

```
CGAACCGATCGGATGACCTTTCTGGAGCGCTTGGAAAATCACTACGAAGTCATTCATGGAGGACATTCATCGCCATTT
  R  T  D  R  M  T  F  L  E  R  L  E  N  H  Y  E  V  I  H  G  G  H  S  S  P  F
CGTTCACCTGCCACATATGAGAAATGTTTACAAAAAGTATTTCCCGAATGCAAAGAAAACCCTGGAGGAAGTCATGGA
  R  S  P  A  T  T  E  K  C  L  Q  K  V  F  P  E  C  K  E  N  P  G  G  S  H  G
TAG
  X
```

Genotype ID = PremStop[13F.3F]

Mutation = Overall 5bp insert (G->CATTCA)

```
CGAACCGATCGGATGACCTTTCTGGAGCGCTTGGAAAATCACTACGAAGTCATTCATTCATGGAGGACATTCATCGCC
  R  T  D  R  M  T  F  L  E  R  L  E  N  H  Y  E  V  I  H  S  W  R  T  F  I  A
ATTTCGTTCACCTGCCACATATGA
  I  S  F  T  C  H  I  X
```

Genotype ID = PremStop[1M.2M]

Mutation = Overall 8bp insert (TGGA->ACTTCATCGAAT)

```
CGAACCGATCGGATGACCTTTCTGGAGCGCTTGGAAAATCACTACGAAGTCATTGACTTCATCGAATGGACATTCATC
  R  T  D  R  M  T  F  L  E  R  L  E  N  H  Y  E  V  I  D  F  I  E  W  T  F  I
GCCATTTCGTTCACCTGCCACATATGA
  A  I  S  F  T  C  H  I  X
```

Genotype ID = PremStop[13M.3F]
Mutation = Overall 4bp deleted (GTGGAGGA->CATT)

```
CGAACCGATCGGATGACCTTTCTGGAGCGCTTGGAAAATCACTACGAAGTCATTCATTCATTCATCGCCATTTCGTTC
  R  T  D  R  M  T  F  L  E  R  L  E  N  H  Y  E  V  I  H  S  F  I  A  I  S  F
ACCTGCCACATATGA
  T  C  H  I  X
```

<u>CRISPR/Cas9 mutations leading to an amino acid sequence change</u>

Genotype ID = AAchange[6M.3M]
Mutation = 3bp (TTG) deleted, removes 1 AA, changes 1 AA

```
CGAACCGATCGGATGACCTTTCTGGAGCGCTTGGAAAATCACTACGAAGTCATGGAGGACATTCATCGCCATTTC
  R  T  D  R  M  T  F  L  E  R  L  E  N  H  Y  E  V  M  E  D  I  H  R  H  F
```

Genotype ID = AAchange[13M.4M]
Mutation = 6bp (GTGGAG) deleted, removes 2 AA

```
CGAACCGATCGGATGACCTTTCTGGAGCGCTTGGAAAATCACTACGAAGTCATTGACATTCATCGCCATTTC
  R  T  D  R  M  T  F  L  E  R  L  E  N  H  Y  E  V  I  D  I  H  R  H  F
```

Genotype ID = AAchange[6F.1M]
Mutation = Overall 6bp insert (TG->GGAGGACA), adds 2 AA, changes 2 AA

```
CGAACCGATCGGATGACCTTTCTGGAGCGCTTGGAAAATCACTACGAAGTCATGGAGGACATGGAGGACATTCATCGC
  R  T  D  R  M  T  F  L  E  R  L  E  N  H  Y  E  V  M  E  D  M  E  D  I  H  R
```

<u>Non-edited chromosomes passed through CRISPR/Cas9 editing pipeline</u>

All 7 have wildtype sequence.

Genotype ID = UnEdit[1F.1F]
Genotype ID (following chromosome substitution) = A4-*Ugt86Dd*^{wt}

Genotype ID = UnEdit[2F.4F]

Genotype ID = UnEdit[3M.5F]

Genotype ID = UnEdit[7F.4M]

Genotype ID = UnEdit[11M.3M]

Genotype ID = UnEdit[13F.2M]

Genotype ID = UnEdit[13F.5F]
