# Supplementary material for: Naturally Segregating Variation at Ugt86Dd Contributes to Nicotine Resistance in Drosophila melanogaster
Source: Genetics. 2017 Jul 26;207(1):311–25. doi: 10.1534/genetics.117.300058 (PMC5586381; doi:10.1534/genetics.117.300058)
Supplement: Supplementary file 8 [file 311TableS1.pdf]

Table S1 Phenotypes of multiple *Ugt86Dd* CRISPR/Cas9-derived homozygous third chromosome genotypes.

**Genotype code** [a]	**Mean viability (no-drug)** [b]	**Mean nicotine resistance** [b]	**Difference** [b]	***P*-value** [c]
PremStop[3F.4F]	0.82	0.24	–0.58	< 0.001
PremStop[8F.3M]	0.82	0.43	–0.39	< 0.01
PremStop[1M.2F]	0.69	0.39	–0.30	< 0.01
PremStop[7F.4F]	0.79	0.33	–0.46	< 0.001
PremStop[13M.5F]	0.85	0.52	–0.33	< 0.05
PremStop[19F.4F]	0.74	0.13	–0.61	< 0.001
PremStop[8F.2F]	0.70	0.20	–0.50	< 0.001
PremStop[13M.2M]	0.73	0.36	–0.37	< 0.01
PremStop[6M.4F]	0.75	0.39	–0.36	< 0.01
PremStop[1F.2F]	0.75	0.38	–0.37	< 0.001
PremStop[13F.3F]	0.75	0.52	–0.23	< 0.01
PremStop[1M.2M]	0.74	0.36	–0.38	< 0.001
PremStop[13M.3F]	0.83	0.49	–0.34	< 0.001
AAchange[6M.3M]	0.83	0.48	–0.35	< 0.001
AAchange[13M.4M]	0.83	0.54	–0.29	< 0.001
AAchange[6F.1M]	0.79	0.26	–0.53	< 0.001
UnEdit[1F.1F]	0.77	0.77	0.00	ns
UnEdit[2F.4F]	0.74	0.77	0.03	ns
UnEdit[3M.5F]	0.68	0.71	0.03	ns
UnEdit[7F.4M]	0.74	0.72	–0.02	ns
UnEdit[11M.3M]	0.78	0.75	–0.03	ns
UnEdit[13F.2M]	0.79	0.77	–0.02	ns
UnEdit[13F.5F]	0.77	0.74	–0.03	ns

[a] "PremStop" means the lesion generates a premature stop codon in *Ugt86Dd*, "AAchange" means the lesion alters the amino acid sequence, while "UnEdit" means that the gene was un-edited, but the genotype was otherwise passed through the same injection/crossing scheme as the edited alleles. The numbers in square brackets are arbitrary lab identifiers. File S4 provides the exact sequence of each lesion.

[b] Each genotype was tested across 5 no-drug and 6 nicotine-containing vials, and the values presented are the means across replicate vials. The difference (nicotine minus no-drug) makes clear the extent, and direction of the change between treatments.

[c] Provides the *P*-value associated with a *t*-test comparing the no-drug and nicotine treatments for each genotype.
