# Supplementary material for: Naturally Segregating Variation at Ugt86Dd Contributes to Nicotine Resistance in Drosophila melanogaster
Source: Genetics. 2017 Jul 26;207(1):311–25. doi: 10.1534/genetics.117.300058 (PMC5586381; doi:10.1534/genetics.117.300058)
Supplement: Supplementary file 9 [file 311TableS2.pdf]

Table S2 Estimate of *Ugt86Dd* InDel allele frequency in natural populations of *D. melanogaster*.

SRA accession [a]	Population [a]	Num. autosomes sampled [a]	Read depth [a]	Num. insertion alleles [b]	Num. deletion alleles [c]	Frequency
SRX661832	Florida	78	69	55	2	0.035
SRX661833	Florida	96	42	32	0	0.000
SRX661834	Georgia	102	118	99	1	0.010
SRX661835	S. Carolina	96	99	98	2	0.020
SRX661837	Pennsylvania	110	216	172	0	0.000
SRX661838	Pennsylvania	148	78	61	0	0.000
SRX661839	Pennsylvania	232	29	24	3	0.111
SRX661840	Pennsylvania	66	89	78	1	0.013
SRX661841	Pennsylvania	150	80	63	1	0.016
SRX661842	Pennsylvania	94	85	72	5	0.065
SRX661843	Pennsylvania	100	81	51	3	0.056
SRX661844	Maine	172	105	60	0	0.000
SRX661845	Maine	150	25	15	0	0.000
			**Total**	880	18	0.020

[a] These data are taken directly from Supplemental Table 1 from Bergland et al. (2014).

[b] The number of reads in the raw FASTQ file containing the sequence ATTGTGGAGGACATTCATCG counted by the UNIX grep function. This sequence corresponds to the central 20bp of the insertion allele at the 22bp InDel. Instances of this sequence appearing in both reads of a paired-end sequenced fragment were counted just once.

[c] The number of reads in the raw FASTQ file containing the sequence GAACGAATTCACTTCGTAGT counted by the UNIX grep function. This sequence corresponds to the 10bp of sequence either side of the insertion allele concatenated together. Instances of this sequence appearing in both reads of a paired-end sequenced fragment were counted just once.
